# *PtrA*, *Piz-t*, and a novel minor-effect QTL (*qBR12_3.3–4.4*) collectively contribute to the durable blast-resistance of rice cultivar Tainung 84

**DOI:** 10.1186/s40529-024-00444-w

**Published:** 2024-12-18

**Authors:** Sheng-Shan Wang, Wei-Bin Chang, Ming-Chien Hsieh, Szu-Yu Chen, Dah-Jing Liao, Ching-Ying Liao, Wei-Chiang Shen, Hong-Hua Chen, Chieh-Yi Chen, Yi-Chia Chen, Yueh-Lin Lin, Chih-Wei Tung, Ruey-Shyang Chen, Chia-Lin Chung

**Affiliations:** 1 Tainan District Agricultural Research and Extension Station, Ministry of Agriculture, No. 70, Muchang, Xinhua, Tainan 712009 Taiwan; 2 Department of Plant Pathology and Microbiology, National Taiwan University, No. 1, Sec. 4, Roosevelt Rd., Taipei City, 106319 Taiwan; 3 Department of Agronomy, Chiayi Agricultural Experiment Branch, Agricultural Research Institute, Ministry of Agriculture, No. 2, Minquan Rd., Chiayi City, 600015 Taiwan; 4 Taitung District Agricultural Research and Extension Station, Ministry of Agriculture, No. 675, Chunghua Rd., Sec. 1, Taitung City, 950244 Taiwan; 5 Department of Agronomy, National Taiwan University, No. 1, Sec. 4, Roosevelt Rd., Taipei City, 106319 Taiwan; 6 Department of Biochemical Science and Technology, National Chiayi University, No. 300, Syuefu Rd., Chiayi City, 600355 Taiwan

**Keywords:** Rice blast, *Pyricularia oryzae*, Linkage mapping, Fine mapping, *Ptr* haplotypes, Durable resistance, Partial resistance, Blast nursery

## Abstract

**Background:**

Rice blast caused by *Pyricularia oryzae* is a major threat to rice production worldwide. Tainung 84 (TNG84) is an elite *japonica* rice cultivar developed through the traditional pedigree method. It has maintained superior blast resistance since its release in 2010. This study aimed to investigate the genetic factors underlying the durable resistance of TNG84 in Taiwan.

**Results:**

Quantitative trait locus (QTL) mapping was conducted using 122 F_2_ individuals and F_2:3_ families derived from the cross of TNG84 and a susceptible *japonica* cultivar Tainan 11 (TN11). Using 733 single nucleotide polymorphisms (SNPs) obtained through genotyping-by-sequencing and three *P. oryzae* isolates (D41-2, 12CY-MS1-2, and 12YL-TT4-1) belonging to different physiological races and Pot2 lineages, a major QTL was identified in the region of 52–54 cM (9.54–15.16 Mb) on chromosome 12. Fine-mapping using 21 F_5:6_ recombinants delimited the QTL to a 140.4-kb region (10.78 to 10.93 Mb) containing the known resistance gene *Ptr*. Sequencing analysis indicated that TNG84 carries the resistant *PtrA* allele and TN11 carries the susceptible *PtrD* allele. Investigation of the *Ptr* haplotypes in 41 local *japonica* rice cultivars revealed that eight *PtrA*-containing cultivars (19.5%) consistently exhibited good field resistance in Taiwan from 2008 to 2024. Subsequently, a few F_5:6_ lines (P026, P044, P092, and P167) lacking the resistant *Ptr* allele were observed to exhibit a resistant phenotype against *P. oryzae* 12YL-TT4-1-lab. Trait-marker association analyses using eight F_6:7_ homozygous recombinants, 378 BC_1_F_2_ from P044 backcrossed to TN11, and 180 BC_1_F_2_ from P092 backcrossed to TN11, identified *Piz-t* on chromosome 6 and a new QTL located between 3.3 Mb and 4.4 Mb on chromosome 12 (designated as *qBR12_3.3–4.4*). Analysis of 12 selected BC_1_F_2:3_ lines derived from P044 demonstrated that in the absence of *Ptr* and *Piz-t*, *qBR12_3.3–4.4* alone reduced the disease severity index from approximately 6.3 to 3.9.

**Conclusions:**

*PtrA* is likely the primary gene responsible for the broad-spectrum and durable resistance of TNG84. *Piz-t* confers narrow-spectrum resistance, while *qBR12_3.3–4.4* contributes partial resistance. The discovery of *qBR12_3.3–4.4* has provided a new source of blast resistance, and the markers developed in this study can be utilized in future breeding programs.

**Supplementary Information:**

The online version contains supplementary material available at 10.1186/s40529-024-00444-w.

## Introduction

Rice is one of the important food crops for the global population. Rice blast, caused by *Pyricularia oryzae* (syn. *Magnaporthe oryzae*), leads to brown oval lesions and withered tissues on leaf blade, ligule, stem, leaf sheath, spike neck, rachis, and grain (Ou 1985). This disease can occur in all rice growing stages and leads to yield losses ranging from 10 to 30% annually. In favorable conditions or during epidemics, it might devastate entire rice plants within 15 to 20 days, resulting in more than 50% yield losses or up to 100% (Asibi et al. 2019). In Taiwan, rice is the most important staple food crop, with an annual cultivated area of ∼169,740 hectares (∼23% of the total cultivated land) (Chen et al. 2021). The rice blast mainly occurs in the first crop season in Taiwan due to significant diurnal temperature variations in spring and high humidity during the rainy season. In 2013, Central-Southern and Eastern Taiwan experienced severe rice blast damage, affecting approximately 30,000 hectares and resulting in production losses of 10–40% (Chen et al. 2020). In 2019, warmer temperatures in February and March (2–4 °C higher than average) triggered an earlier outbreak, and the affected area reached 20–40% in Central and Southern Taiwan (Chen and Chen 2020). In terms of disease management, rice blast can be managed by cultural, chemical, and biological control measures. The proper usage of fungicides such as tricyclazole, isoprothiolane, blasticidin S, kasugamycin, edifenphos, and ferimzone at the proper time can control rice blast well (Froyd et al. 1976; Katagiri and Uesugi 1977; Yamaguchi 2004); however, the use of blast-resistant cultivars is considered a more economical and environmentally friendly method for disease management.

The complex genetics underlying blast resistance involves genes conferring complete and partial resistance. Identification and precise mapping of effective resistance genes in the rice genome are fundamental for successful resistance breeding programs. It often employs QTL mapping approaches to detect major and minor resistance quantitative trait loci (QTLs) using marker systems such as restriction fragment length polymorphism (RFLP), simple sequence repeats (SSRs), and single-nucleotide polymorphisms (SNPs) (Devanna et al. 2022). To date, at least 118 blast resistance genes or QTLs have been identified, and 23 resistance genes with ~ 35 different alleles have been cloned: *Pit*, *Pi-37*, *Pi64*, and *Pish*/*Pi-35* on chromosome 1; *Pib* on chromosome 2; *bsr-d1* on chromosome 3; *pi21* and *Pi63* on chromosome 4; *Pi9*/*Pi2* (= *Piz-5*)/*Piz-t*/*Pi50*/*Pigm*/*Pizh*, *Pid2*, *Pid4*, *Pid3-I1*/*Pi25*/*Pid3* on chromosome 6; *Pi36* on chromosome 8; *Pi5* and *Pi56* on chromosome 9; *Pi1*/*Pik*/*Pik-m*/*Pik-p*/*Pi7*, *Pi54rh*/*Pik-h* (= *Pi54*), *Pik-s*, *Pik-e*, *Pi54of*, *Pb1*, and *Pia*/*PiCO39* on chromosome 11; *Pi-ta* and *Ptr* (= *Pi-ta2*) on chromosome 12 (Kalia and Rathour 2019; Simon et al. 2023; Wang et al. 2017). Most of the cloned blast resistance genes encode nucleotide-binding leucine-rich repeat (NLR) proteins. Indirect or direct recognition between NLR and the corresponding Avirulence (Avr) protein of specific *P. oryzae* races can activate a rapid hypersensitive response in the rice cell, effectively preventing the infection and colonization of *P. oryzae* (Kushalappa et al. 2016). On the other hand, quantitative resistance often involves multiple minor-effect genes, providing partial protection by reducing the pathogenesis and disease severity. Quantitative or partial resistance is often effective against a broad range of pathogen races and tends to be more durable over time (Kou and Wang 2010).

Host-pathogen interactions are long-term arms races. To survive on crops with resistance genes, pathogens must evolve and adapt quickly. Large-scale cultivation of the rice cultivar carrying a major-effect resistance gene could cause high selection pressure and the emergence of *P. oryzae* with mutated Avr, resulting in rapid breakdown of the blast resistance (Liu et al. 2010). For instance, Tainan 11 (TN11) has been the most widely grown *japonica* rice cultivar in Taiwan since 2007, accounting for over 50% of the rice cultivation area. It was initially blast-resistant when released in 2004 but became susceptible by 2006 (Chen et al. 2009; Chen and Lo 2016). Many other Taiwan cultivars, such as Tainung 70 (TNG70), Taikeng 8 (TK8), Taikeng 11 (TK11), Taikeng 14 (TK14), and Taikeng 16 (TK16), were also resistant to blast when first released; however, the rapid breakdown of resistance was observed after 1–5 years of large-scale cultivation (Chen et al. 2004). Therefore, continuous efforts in breeding and identifying disease resistance are crucial for effective rice protection. It is also essential to screen rice germplasm with broad-spectrum and durable resistance and to understand the underlying genetic basis.

Tainung 84 (TNG84) is an elite *japonica* cultivar released in Taiwan in 2010. This mid- to late-maturing cultivar has superior agronomic traits including high yield, good grain appearance, good eating quality, low sprouting on panicle, and low incidence of lodging. For rice diseases, TNG84 performs high resistance to rice blast but susceptibility to bacterial leaf blight and sheath blight (Chen et al. 2011). According to inoculation with 1,749 Taiwanese *P. oryzae* isolates collected from 2014 to 2021, TNG84 displayed broad-spectrum resistance to 98.1% of the isolates (Chen et al. 2023). However, the genetic factors controlling the superior and stable resistance in TNG84 remain unclear. This study aimed to investigate the underlying blast resistance genes/QTL in TNG84 by linkage analyses and sequencing. Additionally, we analyzed the haplotypes of the major-effect gene in local commercial cultivars and investigated their correlation with field resistance from 2008 to 2024. The findings will enhance the understanding of durable resistance to rice blast and contribute to future resistance breeding efforts.

## Materials and methods

### Plant materials

An F_2_ population with 122 individuals was created from the cross of TN11 and TNG84 in 2011. The F_2_ seeds were collected from three F_1_ plants. F_2_ individuals and F_2:3_ families were used for the initial QTL analysis. For fine-mapping, three F_4_ lines heterozygous for the region of 9.40-15.16 Mb in chromosome 12 were selfed to produce a segregating F_5_ population. A total of 1,778 F_5_ individuals were screened with molecular markers C12_0939 and C12_1516 (Table S1), then the recombinants that crossed over in between were selfed to generate 21 heterozygous F_5:6_ and 8 homozygous F_6:7_ recombinant lines for the phenotypic analysis. To investigate other blast resistance QTLs in TNG84, two F_6_ lines carrying homozygous TN11 genotype at *Ptr* (line no. P044 and P092) were backcrossed to TN11 to produce two BC_1_F_2_ segregant populations (384 individuals from P044 and 180 individuals from P092). Twelve BC_1_F_2_ individuals from P044 carrying different combinations of homozygous genotypes at the *Pi2/9* locus and C12_0053 to C12_0440 locus (Table S1) were selfed to produce 12 BC_1_F_2:3_ families. Rice genomic DNA was extracted from leaves following the CTAB method (Murray and Thompson 1980) or using the QuickExtract Plant DNA Extraction Solution (Lucigen, USA) following the manufacturer’s protocol.

### Pathogen isolates

Four *P. oryzae* isolates D41-2, 12CY-MS1-2, 12YL-TT4-1, and 12YL-TT4-1-lab were used for inoculation in this study. D41-2 was collected from Chiayi in 2009, while 12CY-MS1-2 and 12YL-TT4-1 were collected from Chiayi and Yunlin in 2012, respectively. Previous reports showed that *P. oryzae* D41-2 was incompatible to Lijiangxintuanheigu (LTH)-derived International Rice Research Institute-bred blast-resistant lines (IRBLs) containing *Ptr*,* Pi20*,* Pik-m*,* Pik-p*,* Pik*, and *Pi2*; 12CY-MS1-2 was incompatible to IRBLs containing *Ptr*,* Pi20*,* Pik-m*,* Pik-h*,* Pik-p*,* Pik*,* Pi2*,* Pi11*,* Pi9*,* Pi7*,* Pi12*,* Pish*,* Piz*, and *Pi-ta* (= *Pita*); *P. oryzae* 12YL-TT4-1 was incompatible to IRBLs containing *Ptr*,* Pi20*,* Pik-m*,* Pik-h*,* Pik-p*,* Pik*,* Pi2*,* Pi11*,* Pi9*,* Pi7*,* Pi1*,* Pi12*, and *Pib* (Chen et al. 2015; Shih et al. 2017). *P. oryzae* 12YL-TT4-1-lab is a laboratory isolate obtained by Shih et al. (2017) through tissue isolation from an infected LTH leaf inoculated with 12YL-TT4-1. Due to experimental errors or other unknown factors, 12YL-TT4-1-lab was later found to differ from the original isolate. 12YL-TT4-1-lab was tested on IRBLs containing *Pi2*,* Pi9*,* Piz*, and *Piz-t*, and it exhibited incompatibility to *Pi2*,* Pi9*, and *Piz-t* (Shih et al. 2017). All four isolates were incompatible to TNG84 and compatible to TN11, so they are suitable for identifying the resistant factors in TNG84.

The genetic diversity of D41-2, 12CY-MS1-2, and 12YL-TT4-1 was analyzed using Pot2 fingerprinting. The widely used laboratory strain Guy11 was included as the control. The primers Pot2-1 (5'-CGGAAGCCCTAAAGCTGTTT-3') and Pot2-2 (5'-CCCTCATTCGTCACACGTTC-3') targeting the end of Pot2 transposon were used for repetitive element-based PCR. Each PCR reaction contained 12.5 µl *Taq* DNA Polymerase 2× Master Mix RED (Ampliqon, Denmark), 1 µl of 10 µM Pot2-1, 1 µl of 10 µM Pot2-2, and 100 ng template DNA. The total volume was adjusted to 25 µl using ddH_2_O. PCR was conducted with the following thermal cycling parameters: 95 °C for 2.5 min; four cycles of 1 min at 94 °C, 1 min at 62 °C, and 10 min at 65 °C; 26 cycles of 30 s at 94 °C, 1 min at 62 °C, and 10 min at 65 °C; 15 min at 65 °C. PCR products were electrophoresed in a 1.5% agarose gel in 0.5× Tris-borate-EDTA buffer then stained with ethidium bromide (Syauqi et al. 2022).

### Evaluation of disease resistance

To identify blast resistance genes/QTLs, plant materials derived from TN11 x TNG84 were inoculated with *P. oryzae* D41-2, 12CY-MS1-2, 12YL-TT4-1, and 12YL-TT4-1-lab following the method modified from Lin et al. (2018). Rice seeds were disinfected with 1% bleach for 15 min and washed three times with water. Subsequently, seeds were immersed in water for two days and put in the oven at 35 °C to promote germination for one day. The germinated seeds were sown in 2-mm Akadama soil and cultivated in the incubator (28/25°C day/night temperature, 16/8-h light/dark photoperiod). After 7 and 21 days, plants were fertilized with a 500× dilution of HYPONeX No.5 (HYPONEX Corp., USA). The inoculation was conducted about three weeks post sowing (3- to 4-leaf stage). To prepare the inoculum, *P. oryzae* was cultured on oat meal agar at 26 °C in a 12/12-h light/dark photoperiod for two weeks. Conidia were collected using 0.05% Tween 20 (Sigma, USA) and filtered by sterilized double-layered cheesecloth. The concentration of spore suspension was calculated using a hemocytometer and adjusted to 2 × 10^5^ spores/ml. The rice seedlings were evenly sprayed with the spore suspension using an airbrush at 20 psi and then incubated in the plastic box with 100% relative humidity at 25 °C in a dark incubator for 36 h. Afterwards, the plants were kept under 80% relative humidity at 28/26°C day/night temperature and 16/8-h light/dark photoperiod. After 7 days post inoculation (dpi), the disease severity index (DSI) for leaf blast was rated for each seedling based on a 0 to 9 scale (IRRI 2014), with score(s) 0: high resistant (HR); 1–3: resistant (R); 4–5: moderate resistant (MR); 6: moderate susceptible (MS); 7–8: susceptible (S); and 9: high susceptible (HS).

Forty-one Taiwanese *japonica* cultivars and Lomello were evaluated for blast resistance in paddy nurseries at the Chiayi Agricultural Experiment Branch, Taiwan Agricultural Research Institute and Guanshan, Taitung in the first crop season from 2008 to 2024. These cultivars included Hualien 21 (HL21), Hualien 22 (HL22), Hualien 24 (HL24), Hualien 25 (HL25), Kaohsiung 139 (KH139), Kaohsiung 145 (KH145), Kaohsiung 147 (KH147), Miaoli 1 (ML1), Miaoli 2 (ML2), Taichung 192 (TC192), Taichung 194 (TC194), Taichung 196 (TC196), Taikeng 2 (TK2), Taikeng 4 (TK4), TK8, Taikeng 9 (TK9), TK11, TK14, TK16, Taikeng Glutinous 1 (TKN1), Taikeng Glutinous 3 (TKN3), TN11, Tainan 13 (TN13), Tainan 16 (TN16), Tainung 67 (TNG67), TNG70, Tainung 71 (TNG71), Tainung 73 (TNG73), Tainung 74 (TNG74), Tainung 75 (TNG75), Tainung 77 (TNG77), Tainung 78 (TNG78), Tainung 79 (TNG79), Tainung 81 (TNG81), Tainung 82 (TNG82), TNG84, Taitung 30 (TT30), Taitung 33 (TT33), Taitung 35 (TT35), Taitung Glutinous 31 (TTN31), and Taoyuan 3 (TY3). The experimental designs of the paddy blast nurseries were as described by Chen et al. (2021). DSI for leaf blast was rated according to the abovementioned 0 to 9 scale at 30 to 40 days after planting (IRRI 2014).

### QTL mapping using F_2_ and F_2:3_ lines of TN11 x TNG84

The F_2:3_ families derived from 122 F_2_ individuals of TN11 x TNG84 were inoculated with *P. oryzae* D41-2, 12CY-MS1-2, and 12YL-TT4-1. For each isolate, the inoculation was conducted in two independent trials, each containing 20 plants per F_2:3_ family. Inoculated plants were rated on an individual basis and the average DSI for each F_2:3_ family was used for QTL mapping. The genotypes of 122 F_2_ individuals, TN11, and TNG84, were analyzed by genotyping-by-sequencing (GBS) (Elshire et al. 2011). GBS libraries were constructed following the protocol developed by Elshire et al. (2011). Library quality was checked by Bio-Rad Experion (Bio-Rad, USA) and 100-bp single-read sequencing was performed on Illumina Hiseq 2000 at National Yang-Ming University VYM Genome Research Center (Taipei, Taiwan). GBS data were analyzed by de-multiplexing, variant detection, and SNP filtering using TASSEL 3.0 Genotyping by Sequencing Pipeline (Glaubitz et al. 2014). Raw reads were mapped to the Nipponbare reference genome sequences IRGSP-1.0 (Kawahara et al. 2013) using CLC Genomics Workbench 6.5 (QIAGEN, Denmark). To improve data accuracy, only one SNP with the lowest missing rate was retained per 10 kb, and the SNPs with missing rates > 30% were filtered out. After filtering, 733 SNPs were retained for further linkage analysis. Genetic map construction was conducted using R/qtl (Broman et al. 2003). QTL detection was conducted using composite interval mapping (CIM) in QGene Version 4.3.6 (Joehanes and Nelson 2008), with the threshold determined based on 1,000 permutations at a 5% significance level.

### Fine-mapping of the major-effect candidate QTL

To facilitate the marker development for fine-mapping, double digest restriction site-associated DNA sequencing (ddRADseq) was conducted for TN11 and TNG84 to acquire more SNPs (Peterson et al. 2012). The ddRAD sequencing libraries were constructed and 150-bp paired-end sequencing was performed on Illumina HiSeq X-Ten next-generation sequencing platform at Genomics (Taipei, Taiwan). Nipponbare reference genome IRGSP1.0 (Kawahara et al. 2013) was used as the reference sequence, and the raw sequences were analyzed by de-multiplexing, trimming, and variant detection in CLC Genomics Workbench v.11.0 (QIAGEN, Denmark). Through ddRADseq analysis, 12 SNPs evenly distributed across the major-effect candidate QTL were selected, and 12 Kompetitive allele-specific PCR (KASP) markers were designed for fine-mapping (Table S1). KASP analysis was performed according to the manufacturer’s protocol, with a 5-µl total reaction volume, on the CFX96 Connect Real-Time PCR Detection System (Bio-Rad, USA).

A total of 1778 F_5_ individuals were genotyped using the border markers C12_0939 and C12_1516, then 109 recombinants were further genotyped using 10 markers within the candidate region. Twenty-one F_5:6_ recombinant lines carrying heterozygous genotypes for different crossover regions were evaluated for resistance by inoculation with *P. oryzae* D41-2, 12CY-MS1-2, and 12YL-TT4-1-lab. TNG84 and TN11 were used as the control. The experiment was conducted in three independent trials, each containing 3–16 plants per line for D41-1, and 3–12 plants per line for 12CY-MS1-2 and 12YL-TT4-1-lab.

### Analysis of other resistance genes/QTL in the background

The polymorphisms of TNG84 and TN11 at blast resistance genes/loci *Pik*, *Pi2/9*, *Pi5(t)*, and *Pib* were analyzed using markers. KASP markers targeting *Pi2/9* and *Pi5(t)* were developed in this study (Table S1); the markers targeting *Pib* and *Pik* were developed by Hsu et al. (2017). To examine the genotypes in chromosome 12, five markers upstream of C12_0939 and five markers downstream of C12_1516 were developed using the SNP data from ddRADseq (Table S1). Eight F_6:7_ recombinant lines carrying TN11 alleles at *Ptr* were genotyped using these 10 markers. The resistance of these lines was evaluated by inoculation with *P. oryzae* 12YL-TT4-1-lab. The experiment was conducted in two independent trials, each containing 8–12 plants per line.

A population of 378 BC_1_F_2_ individuals from P044 (F_6_) backcrossed with TN11 was used for QTL mapping. The population was genotyped using the markers targeting three co-segregated segments (C12_0053 to C12_0440, C12_1652, and *Piz-t*) and evaluated for resistance by inoculation with *P. oryzae* 12YL-TT4-1-lab (methods as described above). The markers located in 0.53–16.5 Mb of chromosome 12 were used to construct a genetic map using R/qtl (Broman et al. 2003). Haley-Knott regression (method = ‘hk’) in the R/qtl software was conducted for QTL analysis. To validate the minor-effect QTL in the ‘C12_0053 to C12_0440’ region, a population of 180 BC_1_F_2_ individuals from P092 (F_6_) backcrossed with TN11 was used for QTL mapping. This population was homozygous for TN11 at the *Pi2/9* locus but segregating for the ‘C12_0053 to C12_0440’ region. Genotyping, resistance evaluation, and QTL analysis were conducted as described above. The MSU (Michigan State University) Rice Genome Annotation Project Release 7 was used to predict candidate genes in the QTL region (Kawahara et al. 2013; http://rice.plantbiology.msu.edu/). The QTL effects were further examined by inoculating 12 BC_1_F_2:3_ lines with homozygous TN11 or TNG84 genotypes in the candidate regions (*Pi2/9* and the ‘C12_0053 to C12_0440’ loci). The experiment was conducted in two independent trials, each containing 10 plants per line. Differences between each BC_1_F_2:3_ line and TN11 were analyzed by two-tailed Student’s *t*-test.

### Resistance gene sequencing

The genotypes of TNG84 and TN11 at *Ptr* and *Pi2/9* loci were analyzed by PCR amplification and Sanger sequencing. The primers designed for *Ptr* sequencing are listed in Table S2. The sequencing of *Pi2/9* was conducted using Pi2/9-DF1 (5'-CTTGACATCCAAACCGCACC-3') and Pi2/9-DR1 (5'-TAGGCCTAGCCAATTTTTGCC-3') to confirm the presence of *Pi2/9* locus and then using Pi2/9-F3 (5'-AGTTGTTTGCACATGGTGCTGGATG-3') and Pi2/9-R4 (5'-TCAGCCAGCTTGAGCTGTGCCTATC-3') to verify the haplotype (Xiao et al. 2017). The *Ptr* genotypes in another 39 elite *japonica* rice cultivars in Taiwan (as described above) were also examined. Protein sequences were aligned using MultAlign (Corpet 1988).

## Results

### Evaluation of blast resistance in F2:3 families from the cross of TN11 and TNG84

*P. oryzae* D41-2, 12CY-MS1-2, and 12YL-TT4-1 showed different Pot2 fingerprinting patterns (Fig. S1). The Pot2 patterns of D41-2 and 12CY-MS1-2 were similar, whereas 12YL-TT4-1’s notably differed. D41-2 and 12CY-MS1-2 belonged to a major lineage in Taiwan, and 12YL-TT4-1 belonged to a minor lineage (Ruey-Shyang Chen and Wei-Chiang Shen, pers. comm.). Subsequently, F_2:3_ families were evaluated for resistance to *P. oryzae* D41-2, 12CY-MS1-2, and 12YL-TT4-1. Average DSI scores of the F_2_ individuals and F_2:3_ families are shown in Table S3.

### Genetic map construction and QTL mapping

**Fig. 1:**
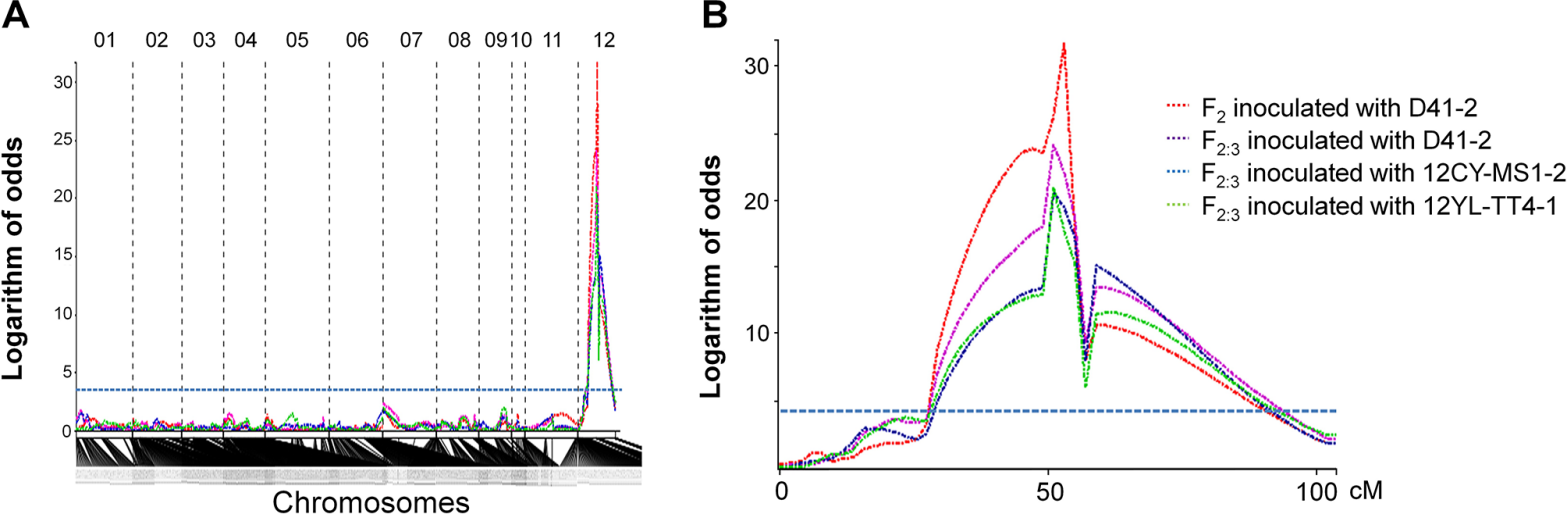
Mapping of blast resistance loci in the F_2_ population and F_2:3_ families of Tainan 11 (TN11) x Tainung 84 (TNG84). (**A**) Logarithm of the odds (LOD) scores on 12 chromosomes. (**B**) LOD scores on chromosome 12. *Pyricularia oryzae* isolates D41-2, 12CY-MS1-2, and 12YL-TT4-1 were used for inoculation. Horizontal line: LOD threshold = 4.34 for F_2_ inoculated with D41-2 (95% confidence level based on 1,000 permutations)

TN11, TNG84, and 122 F_2_ individuals were genotyped using the GBS strategy. A total of ~ 313 million 100-bp single-end reads were obtained, from which 7,742 SNPs were detected. After filtering, 733 reliable SNPs were retained for genetic map construction (Fig. S2). The total length of 12 chromosomes was 1521.6 cM, and the size of each chromosome ranged from 37.7 to 179.5 cM. The map had a high density with an average marker density of 2.11 cM. Chromosome 11 had the largest gap with a distance of 66.6 cM between two adjacent SNPs. For all three *P. oryzae* isolates, linkage analysis using the phenotypes of F_2_ population and F_2:3_ families identified a major QTL in the region of 52–54 cM (9,547,082−15,160,145 bp) on chromosome 12. Logarithm of the odds (LOD) peaks were 31.7, 24.1, 20.6, and 21.0 for F_2_ inoculated with D41-2, F_2:3_ inoculated with D41-2, F_2:3_ inoculated with 12CY-MS1-2, and F_2:3_ inoculated with 12YL-TT4-1, respectively (Fig. 1). The LOD thresholds were 4.34, 3.95, 4.04, and 3.94 for F_2_ inoculated with D41-2, F_2:3_ inoculated with D41-2, F_2:3_ inoculated with 12CY-MS1-2, and F_2:3_ inoculated with 12YL-TT4-1, respectively.

### Fine-mapping and Sanger sequencing revealed the major blast resistance gene *Ptr*

**Fig. 2:**
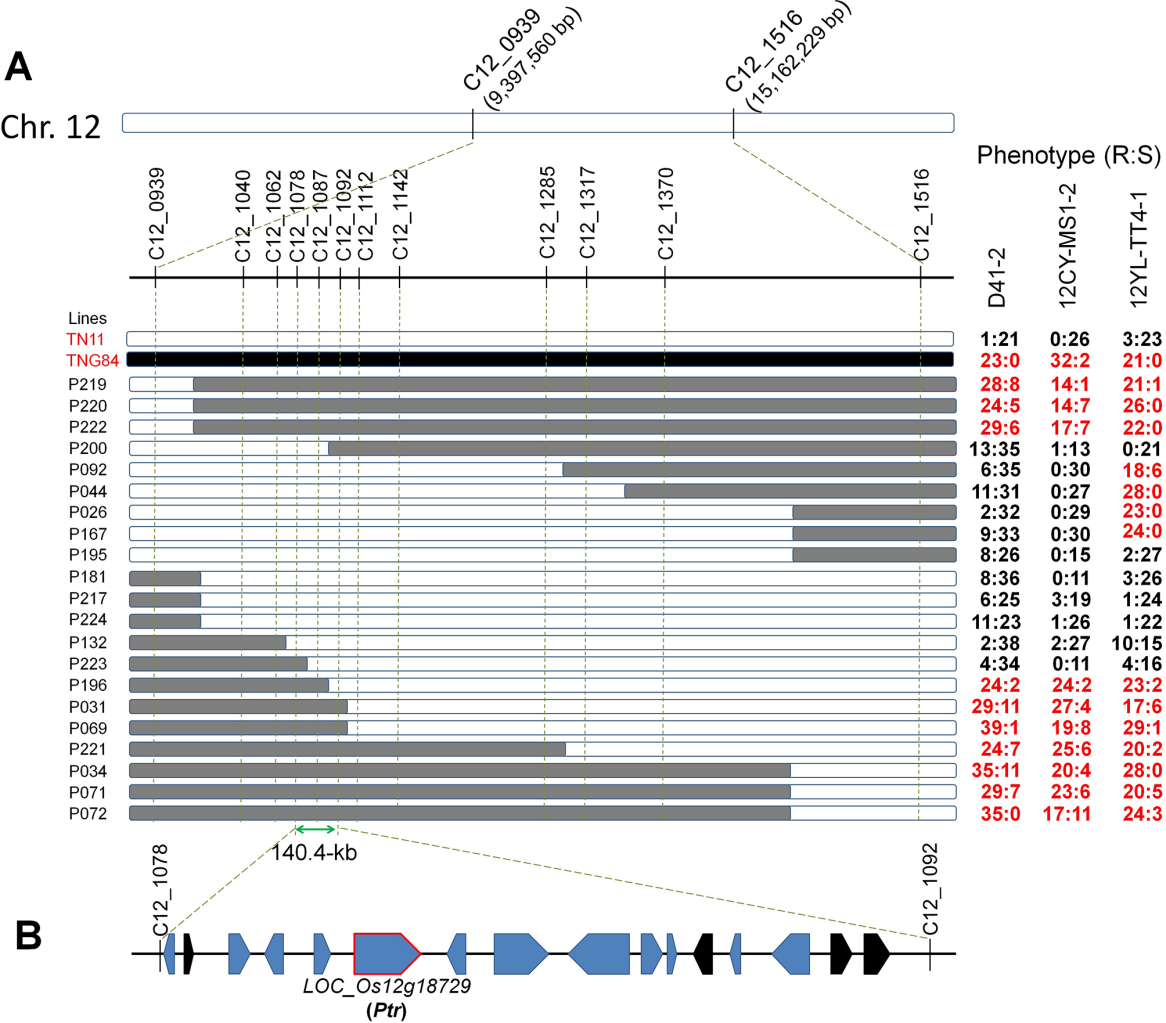
Fine-mapping of the blast resistance QTL on chromosome 12. (**A**) A high-resolution map delimited the QTL to a 140.4-kb region between the C12_1078 and C12_1092 markers. White bars: homozygous Tainan 11 (TN11) chromosome segment; black bars: homozygous Tainung 84 (TNG84) chromosome segment; and grey bars: heterozygous segment. Phenotypes of TN11, TNG84, and 21 F_5:6_ recombinants inoculated with *Pyricularia oryzae* isolates D41-2, 12CY-MS1-2, and 12YL-TT4-1-lab are shown on the right of the bars, with disease severity index scores 0–3 considered resistant (R) and 4–9 considered susceptible (S). The phenotypic data in red represent that most of the inoculated plants were resistant. (**B**) Twelve putative genes and 4 transposon elements were annotated in the candidate QTL region. Blue and black blocks indicate the putative genes and transposon elements predicted in MSU (Michigan State University) Rice Genome Annotation Project Release 7, respectively

To identify the resistance gene on chromosome 12, 21 F_5:6_ recombinants were selected based on the positions of recombination breakpoints, and their phenotypes against three rice blast isolates were evaluated (Fig. 2A). The 21 recombinant lines showed consistent resistance or susceptibility reactions to D41-2 and 12CY-MS1-2. By comparing the resistance and susceptibility phenotypes of the lines possessing different fragments, particularly the lines P196 (R), P200 (S), and P223 (S), the resistance QTL was localized between the markers C12_1078 and C12_1092. According to the MSU Rice Genome Annotation Project Release 7 database (Kawahara et al. 2013; http://rice.plantbiology.msu.edu/), this ~ 140.4-kb region contains four transposon elements and 12 putative genes, including the known blast resistance gene *Ptr* (Fig. 2B) (Zhao et al. 2018). Sanger sequencing analysis revealed that TNG84 carries the same allele as the resistant variety Katy and TN11 carries the same allele as the susceptible variety Amena. Therefore, *Ptr* was recognized as the causal gene for resistance in TNG84 against all three isolates.

### Identification of additional loci associated with the resistance phenotype against *P. oryzae* 12YL-TT4-1-lab

**Fig. 3:**
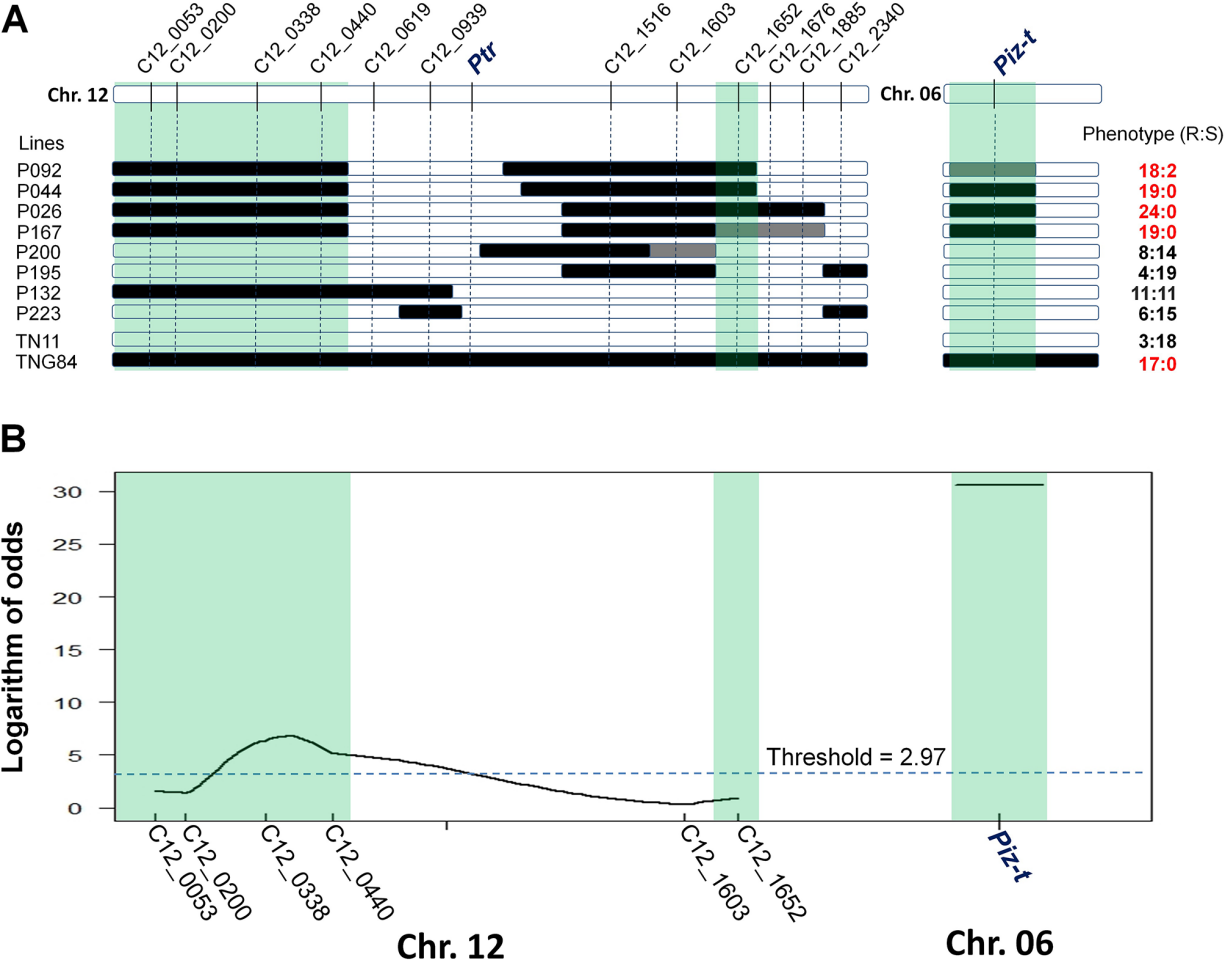
Identification of additional QTLs associated with the resistance against *Pyricularia oryzae* 12YL-TT4-1-lab. (**A**) Graphical genotypes and phenotypes of eight F_6:7_ recombinant lines. Three co-segregated genomic regions (C12_0053 to C12_0440, C12_1652, and *Piz-t*) are shaded. White bars: homozygous Tainan 11 (TN11) chromosome segment; black bars: homozygous Tainung 84 (TNG84) chromosome segment; and grey bars: heterozygous segment. Phenotypes from inoculation with *P. oryzae* isolate 12YL-TT4-1-lab are shown on the right of the bars, with disease severity index scores 0–3 considered resistant (R) and 4–9 considered susceptible (S). The phenotypic data in red represent that most of the inoculated plants were resistant. (**B**) QTL mapping using a BC_1_F_2_ population derived from the F_5:6_ line P044. The horizontal line represents the logarithm of odds threshold at 95% confidence level based on 1,000 permutations

Although the resistance and susceptibility phenotypes from the inoculation results of 12YL-TT4-1-lab were similar to those of D41-2 and 12CY-MS1-2, it was found that four F_5:6_ lines (P092, P044, P026, and P167) that do not carry the resistant *Ptr* allele displayed a resistant phenotype against 12YL-TT4-1-lab (Fig. 2A). This indicated that in addition to *Ptr*, TNG84 should carry other resistance QTLs to 12YL-TT4-1-lab. To analyze whether other regions on chromosome 12 may confer resistance to 12YL-TT4-1-lab, five markers upstream of C12_0939 and five markers downstream of C12_1516 were developed, and trait-marker association analysis was conducted using eight F_6:7_ homozygous recombinant lines without *Ptr* (Fig. 3A). The results revealed that the regions of C12_0053 to C12_0440 and C12_1652 co-segregated with the resistance phenotype against 12YL-TT4-1-lab (see next paragraph for subsequent analyses for C12_0053 to C12_0440 and C12_1652).

To understand whether resistance genes at *Pik*, *Pi2/9*, *Pi5(t)*, and *Pib* are also involved in the resistance against 12YL-TT4-1-lab, we analyzed TNG84 and TN11 using markers targeting these loci. The results showed that TNG84 and TN11 are only polymorphic at the *Pi2/9* locus, excluding the possibility of *Pib*, *Pik*, and *Pi5(t)* resistance. Analysis of the eight F_6:7_ recombinant lines with the *Pi2/9* marker showed that the four resistant lines carried homozygous or heterozygous TNG84 genotypes at the *Pi2/9* locus, whereas the remaining four susceptible lines carried homozygous TN11 genotypes (Fig. 3A). Sanger sequencing analysis revealed that TNG84 allele at the *Pi2/9* locus was identical to *Piz-t* (Genbank accession no.: DQ352040). No product could be amplified from TN11 using primers Pi2/9-DF1 and Pi2/9-DR1, so TN11 may not contain the *Pi2/9* locus. Therefore, *Piz-t* is associated with the resistance to 12YL-TT4-1-lab.

### Identification and validation of a minor-effect QTL in Chr. 12

QTL mapping using a BC_1_F_2_ population derived from P044 and the markers targeting the three co-segregated segments (C12_0053 to C12_0440, C12_1652, and *Piz-t*) revealed that *Piz-t* and a novel QTL located in the C12_0338 to C12_0440 region (designated as *qBR12_3.3–4.4*) were associated with resistance (Fig. 3B; Table 1). *Piz-t* was the major-effect gene explaining 37.3% of phenotypic variance (LOD = 30.6) in the BC_1_F_2_ population, whereas the other minor-effect QTL explained only 13.6% (LOD = 6.8).

The partial resistance of *qBR12_3.3–4.4* was validated in a segregating population without *Piz-t*. QTL analysis of the BC_1_F_2_ population derived from P092 showed that C12_0338 and C12_0440 were significantly associated with resistance (Fig. S3A). Average DSI scores of homozygous TN11, heterozygous, and homozygous TNG84 genotypes at C12_0338 were 5.9, 3.9, and 4.0, respectively (Fig. S3B). The significant difference between TN11 and the other two genotypes indicated that TNG84 has a single dominant genetic factor in the C12_0338 ~ C12_0440 region. According to the MSU Rice Genome Annotation Project Release 7, 129 genes and 39 transposon elements were annotated in the 1.02-Mb interval of *qBR12_3.3–4.4*. Among these, seven disease resistance-related genes, *LOC_Os12g06920*, *LOC_Os12g07060*, *LOC_Os12g07590*, *LOC_Os12g07820*, *LOC_Os12g07830*, *LOC_Os12g07950*, and *LOC_Os12g08180* were identified (Table 2).

**Fig. 4:**
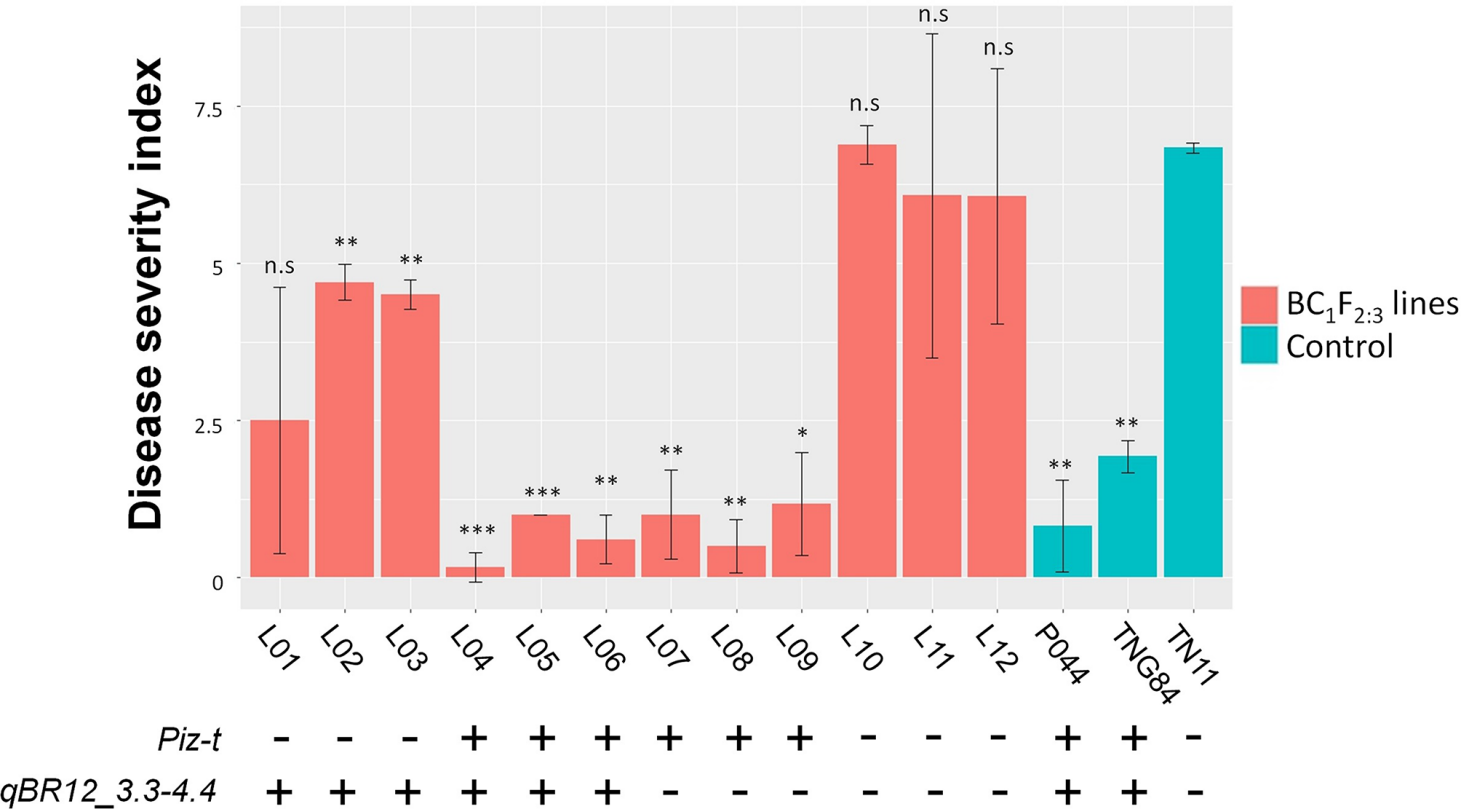
Evaluation of resistance against *Pyricularia oryzae* 12YL-TT4-1-lab in Tainan 11 (TN11), Tainung 84 (TNG84), the F_5:6_ line P044, and 12 BC_1_F_2:3_ lines (L01 to L12). The BC_1_F_2:3_ lines carry different genotypes at *Piz-t* and *qBR12_3.3–4.4*. +: homozygous TNG84 genotypes. -: homozygous TN11 genotypes. Data are mean ± standard deviation. Differences between each line and TN11 were analyzed by two-tailed Student’s *t* test. *, **, and *** indicate *P* < 0.05, 0.01, and 0.001, respectively. n.s indicates not significant

The QTL effects were further analyzed using 12 BC_1_F_2:3_ lines (results in Fig. 4). The lines with homozygous TN11 genotypes at the *Pi2/9* locus and *qBR12_3.3–4.4* were the most susceptible (DSI = 6.1–6.9). The lines carrying homozygous TN11 genotypes at the *Pi2/9* locus but homozygous TNG84 genotypes at the *qBR12_3.3–4.4* were moderately susceptible (DSI = 2.5–4.7). The lines carrying homozygous TNG84 genotypes at the *Pi2/9* locus (i.e., *Piz-t*), no matter with homozygous TN11 or TNG84 genotypes at the *qBR12_3.3–4.4*, exhibited high resistance (DSI < 1.2).

**Table 1:** Mapping of resistance QTL against *P. Oryzae* 12YL-TT4-1-lab in the BC_1_F_2_ population derived from the F_5:6_ line P044

QTL/Gene	Chr.^a^	Marker interval	Physical position (Mb)^b^	LOD^c^	*R* ^2 d^
*Piz-t*	6	Pi2/9	10.4	30.6	37.3
*qBR12_3.3–4.4*	12	C12_0338 to C12_0440	3.3–4.4	6.8	13.6

^a^ Chromosome

^b^ The genomic position is based on the IRGSP-1.0 reference genome

^c^ Logarithm of odds score

^d^ The percentage of phenotypic variance explained by the QTL

**Table 2:** Disease resistance-related candidate genes in the *qBR12_3.3–4.4* region

Gene ID^a^	Physical position (bp)^a^	Putative function
*LOC_Os12g06920*	3,375,469-3,381,282	NLR disease resistance protein, putative, expressed
*LOC_Os12g07060*	3,455,589-3,463,158	OsDjA11 - chaperone protein dnaJ, putative, expressed
*LOC_Os12g07590*	3,792,110-3,797,497	OsPTP1 - protein-tyrosine phosphatase domain containing protein, expressed
*LOC_Os12g07820*	3,955,968-3,959,805	OsAPx6 - stromal ascorbate peroxidase encoding gene 5,8, expressed
*LOC_Os12g07830*	3,960,512-3,964,001	OsAPx5 - stromal ascorbate peroxidase encoding gene 5,8, expressed
*LOC_Os12g07950*	4,032,212-4,036,968	OsSRT2 - transcriptional regulator Sir2 family protein, putative, expressed
*LOC_Os12g08180*	4,178,415-4,182,634	SPL36 - receptor-like protein kinase 2 precursor, putative, expressed

^a^ MSU (Michigan State University) Rice Genome Annotation Project Release 7 was used as the reference genome

### Field resistance and *Ptr* haplotypes of *japonica* rice cultivars in Taiwan

**Fig. 5:**
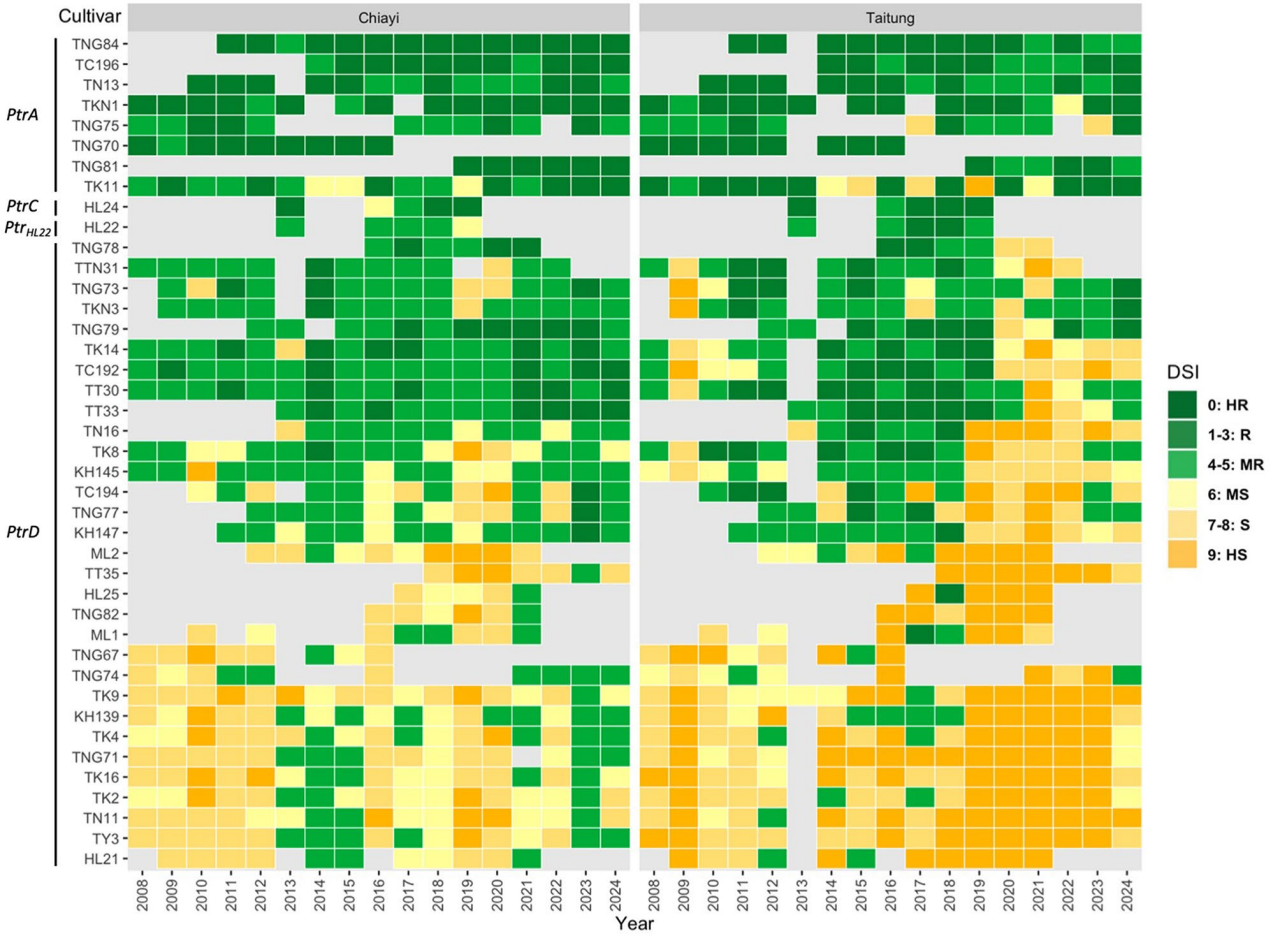
Field resistance and *Ptr* haplotypes of 41 *japonica* rice cultivars in Taiwan. Leaf blast resistance was evaluated in Chiayi and Taitung paddy blast nurseries in the first crop season from 2008 to 2024. Disease severity index (DSI) score was rated according to a 0 to 9 scale at 30 and 40 days after planting. Of the data collected from two scoring time points per season, only the higher DSI scores are shown here. DSI = 0 was considered highly resistant (HR); 1–3 resistant (R); 4–5 moderately resistant (MR); 6 moderately susceptible (MS); 7–8 susceptible (S); and 9 highly susceptible (HS). Light grey represents missing data (the cultivar was not tested in the blast nursery). Four *Ptr* haplotypes (*PtrA*, *PtrC*, *PtrD*, and the novel *Ptr*_*HL22*_) were identified according to the exon 4 sequences (Greenwood et al. 2024; Zhao et al. 2018). The 41 cultivars included Tainung 84 (TNG84), Taichung 196 (TC196), Tainan 13 (TN13), Taikeng Glutinous 1 (TKN1), Tainung 75 (TNG75), Tainung 70 (TNG70), Tainung 81 (TNG81), Taikeng 11 (TK11), Hualien 24 (HL24), Hualien 22 (HL22), Tainung 78 (TNG78), Taitung Glutinous 31 (TTN31), Tainung 73 (TNG73), Taikeng Glutinous 3 (TKN3), Tainung 79 (TNG79), Taikeng 14 (TK14), Taichung 192 (TC192), Taitung 30 (TT30), Taitung 33 (TT33), Tainan 16 (TN16), Taikeng 8 (TK8), Kaohsiung 145 (KH145), Taichung 194 (TC194), Tainung 77 (TNG77), Kaohsiung 147 (KH147), Miaoli 2 (ML2), Taitung 35 (TT35), Hualien 25 (HL25), Tainung 82 (TNG82), Miaoli 1 (ML1), Tainung 67 (TNG67), Tainung 74 (TNG74), Taikeng 9 (TK9), Kaohsiung 139 (KH139), Taikeng 4 (TK4), Tainung 71 (TNG71), Taikeng 16 (TK16), Taikeng 2 (TK2), Tainan 11 (TN11), Taoyuan 3 (TY3), and Hualien 21 (HL21)

Leaf blast resistance of 41 Taiwanese *japonica* rice cultivars in Chiayi and Taitung blast nurseries from 2008 to 2024 are shown in Fig. 5. According to the amino acid sequences of exon 4 of *Ptr*, four haplotypes were identified in these cultivars (Fig. S4) (Greenwood et al. 2024; Zhao et al. 2018). TNG84 and another seven cultivars (TC196, TN13, TKN1, TNG75, TNG70, TNG81, and TK11) were found to carry *PtrA*, the same as the resistant variety Katy. In general, these cultivars have maintained MR to R in both Chiayi and Taitung blast nurseries, except that TKN1 showed MS in 2022 in Taitung, TNG75 showed S in 2017 and 2023 in Taitung, and TK11 showed MS to HS in a few years in both nurseries. HL24 carrying *PtrC* exhibited MS to R in Chiayi and MR to R in Taitung. HL22, carrying a novel haplotype (designated *Ptr*_*HL22*_), showed MS to MR in Chiayi and MR to R in Taitung. The remaining 31 cultivars carry the identical susceptible *PtrD* allele as the susceptible varieties Nipponbare and Lomello. The resistance performance of these *PtrD*-containing cultivars varied: 16 cultivars (ML2, TT35, HL25, TNG82, ML1, TNG67, TNG74, TK9, KH139, TK4, TNG71, TK16, TK2, TN11, TY3, and HL21) showed mostly MS to HS, and six cultivars (TN16, TK8, KH145, TC194, TNG77, and KH147) showed R to HS across different years. Despite without a functional resistant *Ptr* allele, nine cultivars including TNG78, TTN31, TNG73, TKN3, TNG79, TK14, TC192, TT30, and TT33 were mostly MR to R (especially in Chiayi), indicating other underlying resistance genes in their genetic backgrounds. However, the resistance of some cultivars (e.g. TK14 and TC192) has become ineffective in Taitung since 2020.

## Discussion

Rice blast is a major threat to rice production worldwide. The rapid evolution and high variability of *P. oryzae* populations in the field can cause the breakdown of rice resistance and make disease control more challenging (Ashkani et al. 2015; Syauqi et al. 2022). In Taiwan, TNG84 is one of the few rice cultivars showing durable resistance against blast. To analyze the genes/QTLs contributing to its durable resistance, we adopted multiple strategies, including QTL mapping to identify chromosomal regions associated with blast resistance, followed by fine mapping to determine the exact locations of causal resistance genes. Through inoculation with different *P. oryzae* isolates, we identified *Ptr* as the primary resistance gene contributing to broad-spectrum resistance in TNG84. Additionally, we discovered that certain lines lacking the *Ptr* allele still exhibited resistance to the isolate 12YL-TT4-1-lab, indicating the presence of other resistance genes/QTLs. Subsequent analyses revealed that *Piz-t* and a novel minor-effect *qBR12_3.3–4.4* in the 3.38 to 4.40 Mb region of chromosome 12 are responsible for the resistance.

*Ptr* (= *Pi-ta2*) and *Pi-ta* (= *Pita*) were identified as closely linked blast resistance genes on chromosome 12 (Bryan et al. 2000; Meng et al. 2020; Zhao et al. 2018). However, a recent study by Xiao et al. (2024) demonstrated that AVR-Pita recognition relies solely on Ptr instead of Pi-ta. Analysis of 3 K rice germplasm revealed a total of 16 *Ptr* haplotypes, determined based on a 12-bp InDel and several nonsynonymous SNPs in the C-terminal (Zhao et al. 2018). The Pi-ta2 varieties Katy and Pi No. 4 carry *PtrA* allele, conferring resistance against all natural variants of AVR-Pita (Xiao et al. 2024; Zhao et al. 2018). The Pi-ta variety K1 carries *PtrB*, conferring resistance against limited AVR-Pita variants (Xiao et al. 2024). In this study, the TNG84 allele of *Ptr* was identified as *PtrA*. *Pi-ta2* (*PtrA*) was considered one of the most effective resistance genes against the *P. oryzae* population in Taiwan, based on artificial inoculation of IRBLs (Chen et al. 2015) and their 2018–2020 field performance (Chen et al. 2021; Syauqi et al. 2022). By analyzing the *Ptr* sequences of 41 Taiwanese cultivars and comparing the *Ptr* haplotypes with the field resistance/susceptibility from 2008 to 2024, this study provided further evidence for the durability of *PtrA* in Taiwan. Unlike most blast genes that encode NLR proteins, *Ptr* encodes a membrane protein with a cytoplasmic armadillo repeat (ARM) domain (Liu et al. 2021; Xiao et al. 2024; Zhao et al. 2018). Some ARM-containing proteins have been associated with hormonal and stress signaling in plants. The ARM domain typically forms a helical structure that mediates protein-protein interactions. In the context of the most frequently observed U-box/ARM (PUB/ARM) domain, the ARM repeats facilitate the recognition and binding of specific substrate proteins that are targeted for ubiquitination (Sharma and Pandey 2016). Nevertheless, the ARM domain and three transmembrane spans of *Ptr* are distinct from any reported protein classes (Xiao et al. 2024), and its molecular mechanism modulating broad-spectrum resistance remains to be elucidated.

In addition to *Ptr*, *Piz-t* was also identified in TNG84, and it functions specifically against the isolate 12YL-TT4-1-lab. Notably, the laboratory isolate 12YL-TT4-1-lab (Shih et al. 2017) was found to differ from the field isolate 12YL-TT4-1 (Chen et al. 2015) due to its incompatibility with *Piz-t*. This explains why *Piz-t* resistance was not detected during the initial QTL mapping using F_2:3_ families inoculated with 12YL-TT4-1. *Pi2/9* is an important blast resistance locus containing a cluster of *NLR* genes in rice chromosome 6, and *Piz-t* is one of the *NLR* resistance genes identified in this locus (Devanna et al. 2022; Simon et al. 2023; Zhou et al. 2006). During *P. oryzae* infection, the effector AvrPiz-t reduces the transcriptional activity and protein accumulation of APIP5, a negative regulator of cell death. The interaction between Piz-t and APIP5 suppresses the effector-triggered necrosis and enhances resistance (Li et al. 2009; Wang et al. 2016). In Taiwan, among blast resistance genes at the *Pi2/9* locus, *Pi2* and *Pi9* conferred broad-spectrum resistance to 83% and 88% of the tested 18 isolates, respectively; *Piz* demonstrated medium-spectrum resistance against 60% of the tested isolates; and *Piz-t* exhibited narrow-spectrum resistance, effectively combating only 10% of the tested isolates (Chen et al. 2015). It was also observed that IRBLzt-T (carrying *Piz-t*) exhibited susceptibility in different rice blast disease-monitoring plots around Taiwan (Shih et al. 2017; Syauqi et al. 2022). Therefore, although *Piz-t* in TNG84 explained most of the phenotypic variation in the BC_1_F_2_ population derived from P044, its resistance has become ineffective to the dominant *P. oryzae* population in Taiwan. To prolong the resistance of TNG84, other effective resistance genes at the *Pi2/9* locus (e.g. *Pi2* or *Pi9*) could be introduced to replace *Piz-t* in the future.

Recent research has increasingly investigated minor-effect QTLs, highlighting their roles in regulating agronomic traits and disease resistance. Although their influence is smaller than that of major genes/QTL, they can provide minor adjustments of traits in rice breeding (Chen et al. 2014; Fukuoka et al. 2012, 2015). The genetics of blast resistance involves both major- and minor-effect resistance QTLs/genes, and their synergistic interaction reduces the selection pressure on specific pathogen races, thereby enhancing the durability of resistance (Jiang et al. 2020). In this study, a novel minor-effect blast resistance QTL (*qBR12_3.3–4.4*) was detected on chromosome 12 with the LOD of 6.8, accounting for 13.6% of the phenotypic variation in the BC_1_F_2_ population derived from P044 (with *Piz-t* and *qBR12_3.3–4.4* co-segregating). In the analysis of BC_1_F_2:3_ lines, this minor-effect QTL demonstrated partial resistance, reducing the DSI from approximately 6.3 to 3.9 in the absence of *Piz-t*. By resistance evaluation in upland blast nurseries, Nguyen et al. (2006) and Zenbayashi-Sawata et al. (2007) identified *Pi35(t)* and *Pi34* controlling partial resistance of rice cultivars Hokkai 188 and Chubu 32, respectively. Chen et al. (2018) also detected significant additive-by-additive epistatic interactions of two major-effect genes (*Pi-ta* and *Pib*) and three minor-effect QTLs, suggesting that stacking minor QTLs can enhance blast resistance. The KASP markers developed in this study can be used to pyramid the beneficial genotype of *qBR12_3.3–4.4* with other resistance genes through marker-assisted selection.

The *qBR12_3.3–4.4* interval identified in this study contains seven genes associated with disease resistance (Table 2). *LOC_Os12g06920* was annotated as an NLR protein and was found to be upregulated in the rice variety Nekken 2 in response to inoculation with *Xanthomonas oryzae* pv. *oryzicola* (Fan et al. 2023). *LOC_Os12g07060* (*OsDjA11*) is homologous to *OsDjA9* and encodes a chaperone DnaJ protein (Xu et al. 2020), which is crucial for maintaining protein homeostasis under stress conditions (Hartl et al. 2011). A previous study indicated that *OsDjA9* positively modulates rice immunity against *P. oryzae* (Xu et al. 2020). *LOC_Os12g07590* (*OsPTP1*) encodes a protein-tyrosine phosphatase that mediates the crosstalk between salicylic acid (SA)-dependent pathogen defense and abscisic acid (ABA)-induced abiotic stress responses (Ueno et al. 2015). *LOC_Os12g07820* (*OsAPx6*) and *LOC_Os12g07830* (*OsAPx5*) are categorized as ascorbate peroxidase (APX) genes, which are critical antioxidative enzymes involved in resistance to both biotic and abiotic stresses in rice (Agrawal et al. 2003; Jiang et al. 2016). *LOC_Os12g07950* (*OsSRT2*) encodes a histone deacetylase, which is a negative regulator of innate immunity against rice pathogens. CRISPR-Cas9 edited *ossrt2* mutants showed broad-spectrum resistance to *P. oryzae*, *Ustilaginoidea virens*, *Rhizoctonia solani*, and *Xanthomonas oryzae* pv. *oryzae* (Chen et al. 2024). *LOC_Os12g08180* (*SPL36*) is predicted to encode a receptor-like protein kinase that enhances the disease resistance response by influencing the expression of defense- and stress-related genes in rice (Rao et al. 2021). Further experiments are needed to clarify the causal gene(s) and the resistance spectrum of *qBR12_3.3–4.4*.

## Conclusions

TNG84 is an elite rice cultivar developed through long-term and repetitive selection in blast nurseries using the traditional pedigree method. It has maintained superior and stable blast resistance since its release in 2010 (Chen et al. 2023). By QTL mapping and fine-mapping using the progeny populations and recombinant lines derived from TNG84 x TN11, two major-effect genes (*PtrA* and *Piz-t*) and a novel minor-effect QTL (*qBR12_3.3–4.4*) were identified in TNG84. Sequencing analysis indicated that among 41 commercial *japonica* cultivars in Taiwan, eight (19.5%) contained the resistant *PtrA* haplotype. In addition to TNG84, the other seven *PtrA*-containing cultivars also continued to show good field resistance in Taiwan. Therefore, *PtrA* is likely the primary gene responsible for the broad-spectrum and durable resistance of TNG84. *Piz-t* contributes narrow-spectrum resistance, while *qBR12_3.3–4.4* contributes partial resistance. The discovery of *qBR12_3.3–4.4* has provided a new source of blast resistance, and the markers developed in this study can be utilized in future breeding programs. Since most Taiwan cultivars do not carry resistant *Ptr* alleles, the introduction of *PtrA* into mainstream commercial cultivars can be beneficial. Strategies such as variety rotation, gene pyramiding, or multiline should be adopted, combined with good implementation of integrated disease management, to avoid rapid resistance breakdown (Mundt 2014).

## Electronic supplementary material

Below is the link to the electronic supplementary material.

Supplementary Material 1

Supplementary Material 2

Supplementary Material 3

Supplementary Material 4

## Data Availability

The datasets used and/or analysed during the current study are available from the corresponding author on reasonable request.

## Abbreviations

APX: Ascorbate peroxidase
ARM: Armadillo
Avr: Avirulence
CIM: Composite interval mapping
CRISPR: Clustered regularly interspaced short palindromic repeats
ddRADseq: Double digest restriction site-associated DNA sequencing
dpi: Days post inoculation
DSI: Disease severity index
GBS: Genotyping-by-sequencing
HL21: Hualien 21
HL22: Hualien 22
HL24: Hualien 24
HL25: Hualien 25
HR: Highly resistant
HS: Highly susceptible
IRBLs: International Rice Research Institute-bred blast-resistant lines
KASP: Kompetitive allele-specific PCR
KH139: Kaohsiung 139
KH145: Kaohsiung 145
KH147: Kaohsiung 147
LOD: Logarithm of the odds
LTH: Lijiangxintuanheigu
ML1: Miaoli 1
ML2: Miaoli 2
MR: Moderately resistant
MS: Moderately susceptible
MSU: Michigan State University
NLR: Nucleotide-binding leucine-rich repeat
QTL: Quantitative trait locus
R: Resistant
RFLP: Restriction fragment length polymorphism
S: Susceptible
SNPs: Single nucleotide polymorphisms
SSRs: Simple sequence repeats
TK4: Taikeng 4
TK8: Taikeng 8
TK9: Taikeng 9
TK11: Taikeng 11
TK14: Taikeng 14
TK16: Taikeng 16
TKN1: Taikeng Glutinous 1
TKN3: Taikeng Glutinous 3
TN11: Tainan 11
TN13: Tainan 13
TN16: Tainan 16
TNG67: Tainung 67
TNG70: Tainung 70
TNG71: Tainung 71
TNG73: Tainung 73
TNG74: Tainung 74
TNG75: Tainung 75
TNG77: Tainung 77
TNG78: Tainung 78
TNG79: Tainung 79
TNG81: Tainung 81
TNG82: Tainung 82
TNG84: Tainung 84
TT30: Taitung 30
TT33: Taitung 33
TT35: Taitung 35
TTN31: Taitung Glutinous 31
TY3: Taoyuan 3

## References

[CR1] Agrawal GK, Jwa NS, Iwahashi H, Rakwal R (2003) Importance of ascorbate peroxidases *OsAPX1* and *OsAPX2* in the rice pathogen response pathways and growth and reproduction revealed by their transcriptional profiling. Gene 322:93–103. 10.1016/j.gene.2003.08.017 14644501 10.1016/j.gene.2003.08.017

[CR2] Ashkani S, Rafii MY, Shabanimofrad M, Miah G, Sahebi M, Azizi P, Tanweer FA, Akhtar MS, Nasehi A (2015) Molecular breeding strategy and challenges towards improvement of blast disease resistance in rice crop. Front Plant Sci 6:886. 10.3389/fpls.2015.00886 26635817 10.3389/fpls.2015.00886 PMC4644793

[CR3] Asibi AE, Chai Q, Coulter JA (2019) Rice blast: a disease with implications for global food security. Agronomy 9(8):451. 10.3390/agronomy9080451

[CR4] Broman KW, Wu H, Sen S, Churchill GA (2003) R/qtl: QTL mapping in experimental crosses. Bioinformatics 19(7):889–890. 10.1093/bioinformatics/btg112 12724300 10.1093/bioinformatics/btg112

[CR5] Bryan GT, Wu KS, Farrall L, Jia Y, Hershey HP, McAdams SA, Faulk KN, Donaldson GK, Tarchini R, Valent B (2000) A single amino acid difference distinguishes resistant and susceptible alleles of the rice blast resistance gene *Pi-ta*. Plant Cell 12:2033–2045. 10.1105/tpc.12.11.2033 11090207 10.1105/tpc.12.11.2033 PMC150156

[CR15] Chen YN, Chen PC (2020) The neglected first inoculum of rice blast pathogen *pyricularia oryze* on the mats of rice seedlings. J Plant Med 62:13–16. 10.6716/JPM.202006_62(2).0002

[CR10] Chen LC, Lo JC (2016) Contribution of the rice cultivar Tainan 11. Tainan Dist Agric Newsl 97:12–15

[CR7] Chen LC, Chen YS, Cheng YH (2004) Test of rice varieties and strains resistant to rice blast in blast nurseries during 1990–2002. Jour Agric Res China 53:269

[CR8] Chen LC, Huang SH, Cheng CH (2009) Review of the screening tests for rice varietal resistance to major diseases and insect pests in Taiwan. In: Proceedings of symposium on achievements and perspectives of rice protection in Taiwan, 161p, 1st (ed), Chiayi Agricultural Experiment Branch, Taiwan Agricultural Research Institute, Chiayi, Taiwan, pp 83–103

[CR9] Chen LC, Liao DJ, Hung SH, Jwo WS, Yen HM, Lo JC, Chen RK (2011) Breeding a new *japonica* rice variety Tainung 84. J Taiwan Agric Res 60:221–238

[CR6] Chen JY, Guo L, Ma H, Chen YY, Zhang HW, Ying JZ, Zhuang JY (2014) Fine mapping of *qHd1*, a minor heading date QTL with pleiotropism for yield traits in rice (*Oryza sativa* L). Theor Appl Genet 127(11):2515–2524. 10.1007/s00122-014-2395-7 25223543 10.1007/s00122-014-2395-7 PMC4209109

[CR11] Chen WL, Shen WC, Chang FY, Chang WB, Yu TH, Lai MH, Liao JY, Wu C, Chung CL (2015) Analysis of blast resistance genes and molecular markers development for the improvement of Taiwan high-quality rice varieties. Plant Pathol Bull 24:225–240. 10.6649/PPB.201512_24(3_4).0005

[CR12] Chen X, Jia Y, Jia MH, Pinson SR, Wang X, Wu B (2018) Functional interactions between major rice blast resistance genes, *Pi-ta* and *Pi-b*, and minor blast resistance quantitative trait loci. Phytopathology 108(9):1095–1103. 10.1094/PHYTO-02-18-0032-R 29658844 10.1094/PHYTO-02-18-0032-R

[CR16] Chen YN, Chen MC, Chen PC (2020) Pathogen diversity and inoculum source of rice blast disease in Taiwan. In: JIRCAS-FFTC International Workshop on Applicable Solutions Against Rice Blast in Asia Proceedings, pp 93–106

[CR14] Chen YC, Hu CC, Chang FY, Chen CY, Chen WL, Tung CW, Shen WC, Wu CW, Cheng AH, Liao DJ, Liao CY, Liu LD, Chung CL (2021) Marker-assisted development and evaluation of monogenic lines of rice cv. Kaohsiung 145 carrying blast resistance genes. Plant Dis 105(12):3858–3868. 10.1094/PDIS-01-21-0142-RE 34181437 10.1094/PDIS-01-21-0142-RE

[CR17] Chen YN, Wu DH, Chen MC, Hsieh MT, Jwo WS, Lin GC, Chen RK, Chou HP, Chen PC (2023) Dynamics of spatial and temporal population structure of *Pyricularia oryzae* in Taiwan. Pest Manag Sci 79(11):4254–4263. 10.1002/ps.7621 37341444 10.1002/ps.7621

[CR13] Chen X, Liu C, Wang H, Liu Q, Yue Y, Duan Y, Wang Z, Zheng L, Chen X, Wang Y, Huang J, Xu Q, Pan Y (2024) *Ustilaginoidea virens*-secreted effector Uv1809 suppresses rice immunity by enhancing OsSRT2-mediated histone deacetylation. Plant Biotechnol J 22(1):148–164. 10.1111/pbi.14174 37715970 10.1111/pbi.14174 PMC10754013

[CR18] Corpet F (1988) Multiple sequence alignment with hierarchical clustering. Nucleic Acids Res 16(22):10881–10890. 10.1093/nar/16.22.10881 2849754 10.1093/nar/16.22.10881 PMC338945

[CR19] Devanna BN, Jain P, Solanke AU, Das A, Thakur S, Singh PK, Kumari M, Dubey H, Jaswal R, Pawar D (2022) Understanding the dynamics of blast resistance in rice-*magnaporthe oryzae* interactions. J Fungi 8(6):584. 10.3390/jof8060584 10.3390/jof8060584 PMC9224618 35736067

[CR20] Elshire RJ, Glaubitz JC, Sun Q, Poland JA, Kawamoto K, Buckler ES, Mitchell SE (2011) A robust, simple genotyping-by-sequencing (GBS) approach for high diversity species. PLoS ONE 6(5):e19379. 10.1371/journal.pone.0019379 21573248 10.1371/journal.pone.0019379 PMC3087801

[CR21] Fang Y, Ding D, Gu Y, Jia Q, Zheng Q, Qian Q, Wang Y, Rao Y, Mao Y (2023) Identification of QTLs conferring resistance to bacterial diseases in rice. Plants (Basel) 12(15):2853. 10.3390/plants12152853 37571006 10.3390/plants12152853 PMC10421440

[CR22] Froyd JD, Paget CJ, Guse LR, Dreikorn BA, Pafford JL (1976) Tricyclazole: a new systemic fungicide for control of *Pyricularia oryzae* on rice. Phytopathology 66:1135–1139. 10.1094/Phyto-66-1135

[CR23] Fukuoka S, Mizobuchi R, Saka N, Suprun I, Matsumoto T, Okuno K, Yano M (2012) A multiple gene complex on rice chromosome 4 is involved in durable resistance to rice blast. Theor Appl Genet 125(3):551–559. 10.1007/s00122-012-1852-4 22446930 10.1007/s00122-012-1852-4 PMC3397134

[CR24] Fukuoka S, Saka N, Mizukami Y, Koga H, Yamanouchi U, Yoshioka Y, Hayashi N, Ebana K, Mizobuchi R, Yano M (2015) Gene pyramiding enhances durable blast disease resistance in rice. Sci Rep 5:7773. 10.1038/srep07773 25586962 10.1038/srep07773 PMC5379001

[CR25] Glaubitz JC, Casstevens TM, Lu F, Harriman J, Elshire RJ, Sun Q, Buckler ES (2014) TASSEL-GBS: a high capacity genotyping by sequencing analysis pipeline. PLoS ONE 9(2):e90346. 10.1371/journal.pone.0090346 24587335 10.1371/journal.pone.0090346 PMC3938676

[CR26] Greenwood JR, Lacorte-Apostol V, Kroj T, Padilla J, Telebanco-Yanoria MJ, Glaus AN, Roulin A, Padilla A, Zhou B, Keller B (2024) Genome-wide association analysis uncovers rice blast resistance alleles of *Ptr* and *Pia*. Commun Biol 7:607. 10.1038/s42003-024-06244-z 38769168 10.1038/s42003-024-06244-z PMC11106262

[CR27] Hartl F, Bracher A, Hayer-Hartl M (2011) Molecular chaperones in protein folding and proteostasis. Nature 475:324–332. 10.1038/nature10317 21776078 10.1038/nature10317

[CR28] Hsu LH, Wang SS, Chen RK (2017) Molecular marker development for rice blast resistance genes and the analysis of *Pik* locus haplotypes in Taiwan rice cultivars. Res Bull Tainan Dist Agric Improv Stn 70:11–23

[CR29] IRRI (2014) Standard evaluation system for rice, 5th edn. International Rice Research Institute, p 57

[CR30] Jiang G, Yin D, Zhao J, Chen H, Guo L, Zhu L, Zhai W (2016) The rice thylakoid membrane-bound ascorbate peroxidase OsAPX8 functions in tolerance to bacterial blight. Sci Rep 6:26104. 10.1038/srep26104 27185545 10.1038/srep26104 PMC4868969

[CR31] Jiang H, Feng Y, Qiu L, Gao G, Zhang Q, He Y (2020) Identification of blast resistance QTLs based on two advanced backcross populations in rice. Rice 13(1):31. 10.1186/s12284-020-00392-6 32488495 10.1186/s12284-020-00392-6 PMC7266886

[CR32] Joehanes R, Nelson JC (2008) QGene 4.0, an extensible Java QTL-analysis platform. Bioinformatics 24(23):2788–2789. 10.1093/bioinformatics/btn523 18940826 10.1093/bioinformatics/btn523

[CR33] Kalia S, Rathour R (2019) Current status on mapping of genes for resistance to leaf-and neck-blast disease in rice. 3 Biotech 9(6):209. 10.1007/s13205-019-1738-0 31093479 10.1007/s13205-019-1738-0 PMC6509304

[CR34] Katagiri M, Uesugi Y (1977) Similarities between the fungicidal action of isoprothiolane and organophosphorus thiolate fungicides. Phytopathology 67:1415–1417

[CR35] Kawahara Y, de la Bastide M, Hamilton JP, Kanamori H, McCombie WR, Ouyang S, Schwartz DC, Tanaka T, Wu J, Zhou S, Childs KL, Davidson RM, Lin H, Quesada-Ocampo L, Vaillancourt B, Sakai H, Lee SS, Kim J, Numa H, Itoh T, Buell CR, Matsumoto T (2013) Improvement of the *Oryza sativa* nipponbare reference genome using next generation sequence and optical map data. Rice 6(1):4. 10.1186/1939-8433-6-4 24280374 10.1186/1939-8433-6-4 PMC5395016

[CR36] Kou Y, Wang S (2010) Broad-spectrum and durability: understanding of quantitative disease resistance. Curr Opin Plant Biol 13:181–185. 10.1016/j.pbi.2009.12.010 20097118 10.1016/j.pbi.2009.12.010

[CR37] Kushalappa AC, Yogendra KN, Karre S (2016) Plant innate immune response: qualitative and quantitative resistance. Crit Rev Plant Sci 35(1):38–55. 10.1080/07352689.2016.1148980

[CR38] Li W, Wang B, Wu J, Lu G, Hu Y, Zhang X, Zhang Z, Zhao Q, Feng Q, Zhang H (2009) The *Magnaporthe oryzae* avirulence gene *AvrPiz-t* encodes a predicted secreted protein that triggers the immunity in rice mediated by the blast resistance gene *Piz-t*. Mol Plant Microbe Interact 22(4):411–420. 10.1094/MPMI-22-4-0411 19271956 10.1094/MPMI-22-4-0411

[CR39] Lin HA, Chen SY, Chang FY, Tung CW, Chen YC, Shen WC, Chen RS, Wu CW, Chung CL (2018) Genome-wide association study of rice genes and loci conferring resistance to *Magnaporthe Oryzae* isolates from Taiwan. Bot Stud 59(1):32. 10.1186/s40529-018-0248-4 30578469 10.1186/s40529-018-0248-4 PMC6303224

[CR40] Liu J, Wang X, Mitchell T, Hu Y, Liu X, Dai L, Wang GL (2010) Recent progress and understanding of the molecular mechanisms of the rice-*magnaporthe oryzae* interaction. Mol Plant Pathol 11(3):419–427. 10.1111/j.1364-3703.2009.00607.x 20447289 10.1111/j.1364-3703.2009.00607.x PMC6640493

[CR41] Liu Z, Zhu Y, Shi H, Qiu J, Ding X, Kou Y (2021) Recent progress in rice broad-spectrum disease resistance. Int J Mol Sci 22(21):11658. 10.3390/ijms222111658 34769087 10.3390/ijms222111658 PMC8584176

[CR42] Meng X, Xiao G, Telebanco-Yanoria MJ, Siazon PM, Padilla J, Opulencia R, Bigirimana J, Habarugira G, Wu J, Li M (2020) The broad-spectrum rice blast resistance (*R*) gene *Pita2* encodes a novel R protein unique from *Pita*. Rice 13(1):19. 10.1186/s12284-020-00377-5 32170462 10.1186/s12284-020-00377-5 PMC7070119

[CR43] Mundt CC (2014) Durable resistance: a key to sustainable management of pathogens and pests. Infect Genet Evol 27:446–455. 10.1016/j.meegid.2014.01.011 24486735 10.1016/j.meegid.2014.01.011 PMC4117828

[CR44] Murray M, Thompson W (1980) Rapid isolation of high molecular weight plant DNA. Nucleic Acids Res 8(19):4321–4325. 10.1093/nar/8.19.4321 7433111 10.1093/nar/8.19.4321 PMC324241

[CR45] Nguyen T, Koizumi S, La T, Zenbayashi K, Ashizawa T, Yasuda N, Imazaki I, Miyasaka A (2006) *Pi35*(t), a new gene conferring partial resistance to leaf blast in the rice cultivar Hokkai 188. Theor Appl Genet 113(4):697–704. 10.1007/s00122-006-0337-8 16838138 10.1007/s00122-006-0337-8

[CR46] Ou SH (1985) Rice diseases, 2nd edn. Commonwealth Mycological Institute, Kew, p 380

[CR47] Peterson BK, Weber JN, Kay EH, Fisher HS, Hoekstra HE (2012) Double digest RADseq: an inexpensive method for de novo SNP discovery and genotyping in model and non-model species. PLoS ONE 7(5):e37135. 10.1371/journal.pone.0037135 22675423 10.1371/journal.pone.0037135 PMC3365034

[CR59] Rao Y, Jiao R, Wang S, Wu X, Ye H, Pan C, Li S, Xin D, Zhou W, Dai G, Hu J, Ren D, Wang Y (2021) *SPL36* encodes a receptor-like protein kinase that regulates programmed cell death and defense responses in rice. Rice 14(1):34. 10.1186/s12284-021-00475-y 33825994 10.1186/s12284-021-00475-y PMC8026784

[CR48] Sharma M, Pandey GK (2016) Expansion and function of repeat domain proteins during stress and development in plants. Front Plant Sci 6:1218. 10.3389/fpls.2015.01218 26793205 10.3389/fpls.2015.01218 PMC4707873

[CR49] Shih YC, Liao DJ, Wu YP, Shen WC, Chang WB, Chen CY, Chung CL (2017) Identification of blast resistance gene *Piz-t* in a rice cultivar Taikeng 14. J Plant Med 59:23–31. 10.6716/JPM.201712_59(4).0004

[CR50] Simon EV, Hechanova SL, Hernandez JE, Li CP, Tülek A, Ahn EK, Jairin J, Choi IR, Sundaram RM, Jena KK (2023) Available cloned genes and markers for genetic improvement of biotic stress resistance in rice. Front Plant Sci 14:1247014. 10.3389/fpls.2023.1247014 37731986 10.3389/fpls.2023.1247014 PMC10507716

[CR51] Syauqi J, Chen RK, Cheng AH, Wu YF, Chung CL, Lin CC, Chou HP, Wu HY, Jian JY, Liao CT (2022) Surveillance of rice blast resistance effectiveness and emerging virulent isolates in Taiwan. Plant Dis 106(12):3187–3197. 10.1094/PDIS-12-21-2806-RE 35581907 10.1094/PDIS-12-21-2806-RE

[CR52] Ueno Y, Yoshida R, Kishi-Kaboshi M, Matsushita A, Jiang CJ, Goto S, Takahashi A, Hirochika H, Takatsuji H (2015) Abiotic stresses antagonize the rice defence pathway through the tyrosine-dephosphorylation of OsMPK6. PLoS Pathog 11(10):e1005231. 10.1371/journal.ppat.1005231 26485146 10.1371/journal.ppat.1005231 PMC4617645

[CR54] Wang R, Ning Y, Shi X, He F, Zhang C, Fan J, Jiang N, Zhang Y, Zhang T, Hu Y (2016) Immunity to rice blast disease by suppression of effector-triggered necrosis. Curr Biol 26(18):2399–2411. 10.1016/j.cub.2016.06.072 27641772 10.1016/j.cub.2016.06.072

[CR53] Wang Bh, Ebbole DJ, Wang Z (2017) The arms race between *Magnaporthe oryzae* and rice: Diversity and interaction of *Avr* and *R* genes. J Integr Agric 16(12):2746–2760. 10.1016/S2095-3119(17)61746-5

[CR55] Xiao G, Borja FN, Mauleon R, Padilla J, Telebanco-Yanoria MJ, Yang J, Lu G, Dionisio-Sese M, Zhou B (2017) Identification of resistant germplasm containing novel resistance genes at or tightly linked to the *Pi2/9* locus conferring broad-spectrum resistance against rice blast. Rice 10:37. 10.1186/s12284-017-0176-z 28779340 10.1186/s12284-017-0176-z PMC5544663

[CR56] Xiao G, Laksanavilat N, Cesari S, Lambou K, Baudin M, Jalilian A, Telebanco-Yanoria MJ, Chalvon V, Meusnier I, Fournier E (2024) The unconventional resistance protein PTR recognizes the *Magnaporthe oryzae* effector AVR-Pita in an allele-specific manner. Nat Plants 10:994–1004. 10.1038/s41477-024-01694-z 38834685 10.1038/s41477-024-01694-z

[CR57] Xu G, Zhong X, Shi Y, Liu Z, Jiang N, Liu J, Ding B, Li Z, Kang H, Ning Y, Liu W, Guo Z, Wang GL, Wang X (2020) A fungal effector targets a heat shock-dynamin protein complex to modulate mitochondrial dynamics and reduce plant immunity. Sci Adv 6(48):eabb7719. 10.1126/sciadv.abb7719 33239288 10.1126/sciadv.abb7719 PMC7688324

[CR58] Yamaguchi I (2004) Overview on the chemical control of rice blast disease. In: Rice Blast, Interaction with rice and control, Proceedings of the 3rd International Rice Blast Conference Springer, pp 1–13

[CR60] Zenbayashi-Sawata K, Fukuoka S, Katagiri S, Fujisawa M, Matsumoto T, Ashizawa T, Koizumi S (2007) Genetic and physical mapping of the partial resistance gene, *Pi34*, to blast in rice. Phytopathology 97(5):598–602. 10.1094/PHYTO-97-5-0598 18943579 10.1094/PHYTO-97-5-0598

[CR61] Zhao H, Wang X, Jia Y, Minkenberg B, Wheatley M, Fan J, Jia MH, Famoso A, Edwards JD, Wamishe Y (2018) The rice blast resistance gene *ptr* encodes an atypical protein required for broad-spectrum disease resistance. Nat Commun 9:2039. 10.1038/s41467-018-04369-4 29795191 10.1038/s41467-018-04369-4 PMC5966436

[CR62] Zhou B, Qu S, Liu G, Dolan M, Sakai H, Lu G, Bellizzi M, Wang GL (2006) The eight amino-acid differences within three leucine-rich repeats between Pi2 and Piz-t resistance proteins determine the resistance specificity to *Magnaporthe Grisea*. Mol Plant Microbe Interact 19(11):1216–1228. 10.1094/MPMI-19-1216 17073304 10.1094/MPMI-19-1216

