# Supplementary material for: PtrA, Piz-t, and a novel minor-effect QTL (qBR12_3.3–4.4) collectively contribute to the durable blast-resistance of rice cultivar Tainung 84
Source: Bot Stud. 2024 Dec 18;65:37. doi: 10.1186/s40529-024-00444-w (PMC11655991; doi:10.1186/s40529-024-00444-w)
Supplement: Supplementary file 4 — Supplementary Material 4 [file 40529_2024_444_MOESM4_ESM.doc]

**
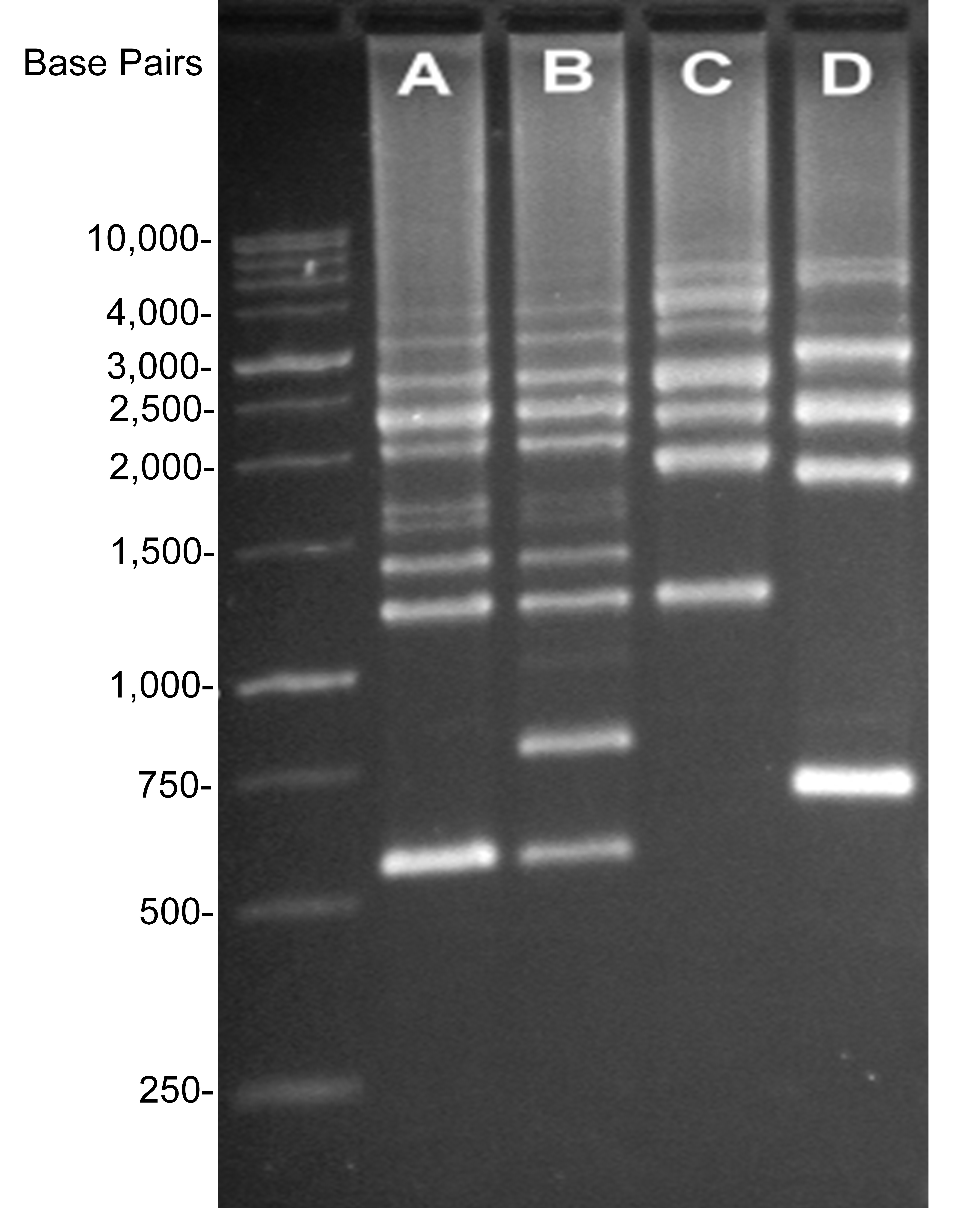
**

**Fig. S1** Pot2 fingerprinting patterns of *Pyricularia oryzae* isolates. (A) D41-2l; (B) 12CY-MS1-2; (C) 12YL-TT4-1; and (D) Guy11. First lane: Omics 1kb DNA RTU Ladder.

**
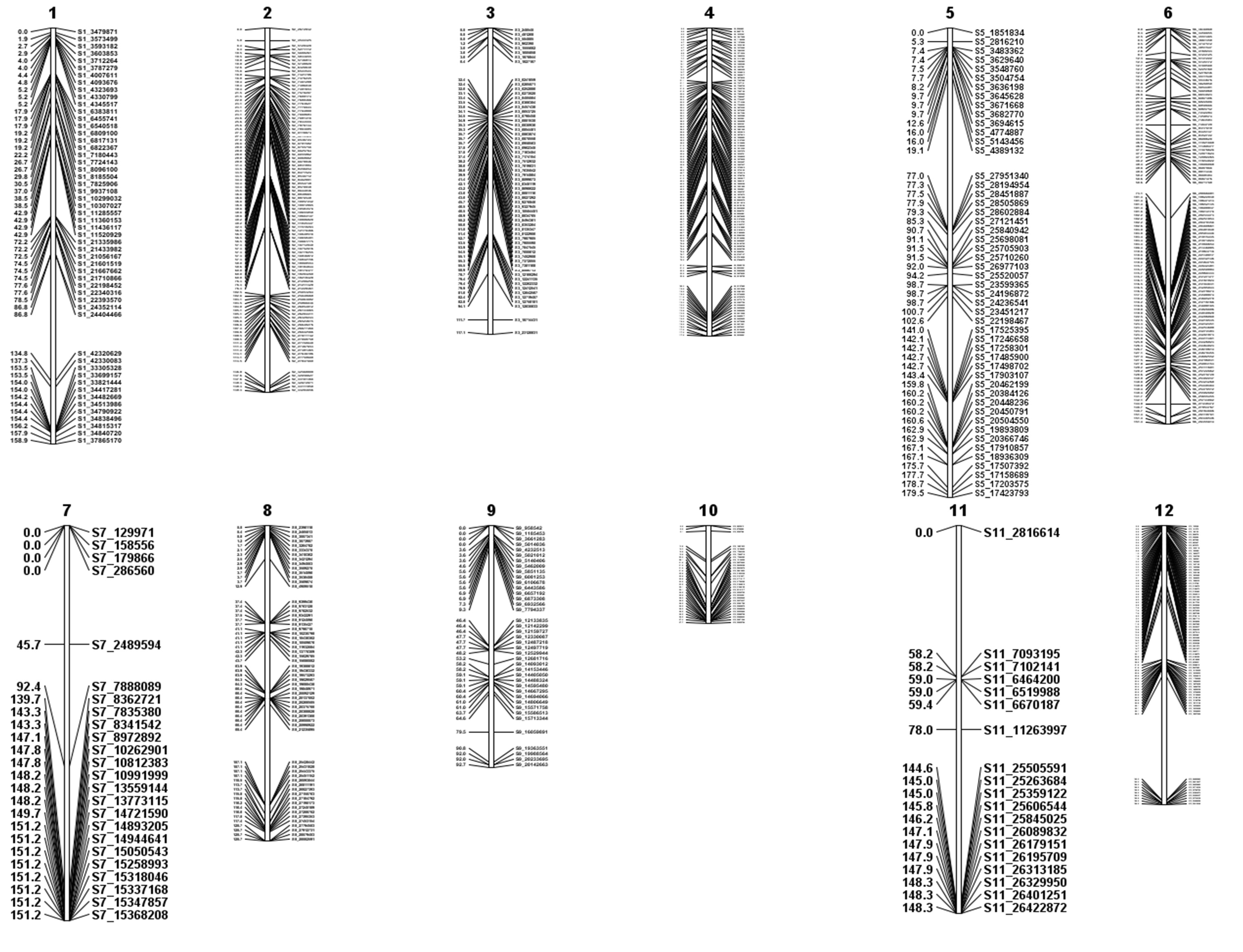
**

**Fig. S2** Genetic map of the F_2_ population of Tainung 84 (TNG84) x Tainan 11 (TN11). The map was constructed using 733 reliable single nucleotide polymorphism (SNPs) obtained through genotyping-by-sequencing. The numbers to the left of the chromosomes represent the genetic map positions; the labels to the right are SNP locus names based on the physical map positions.


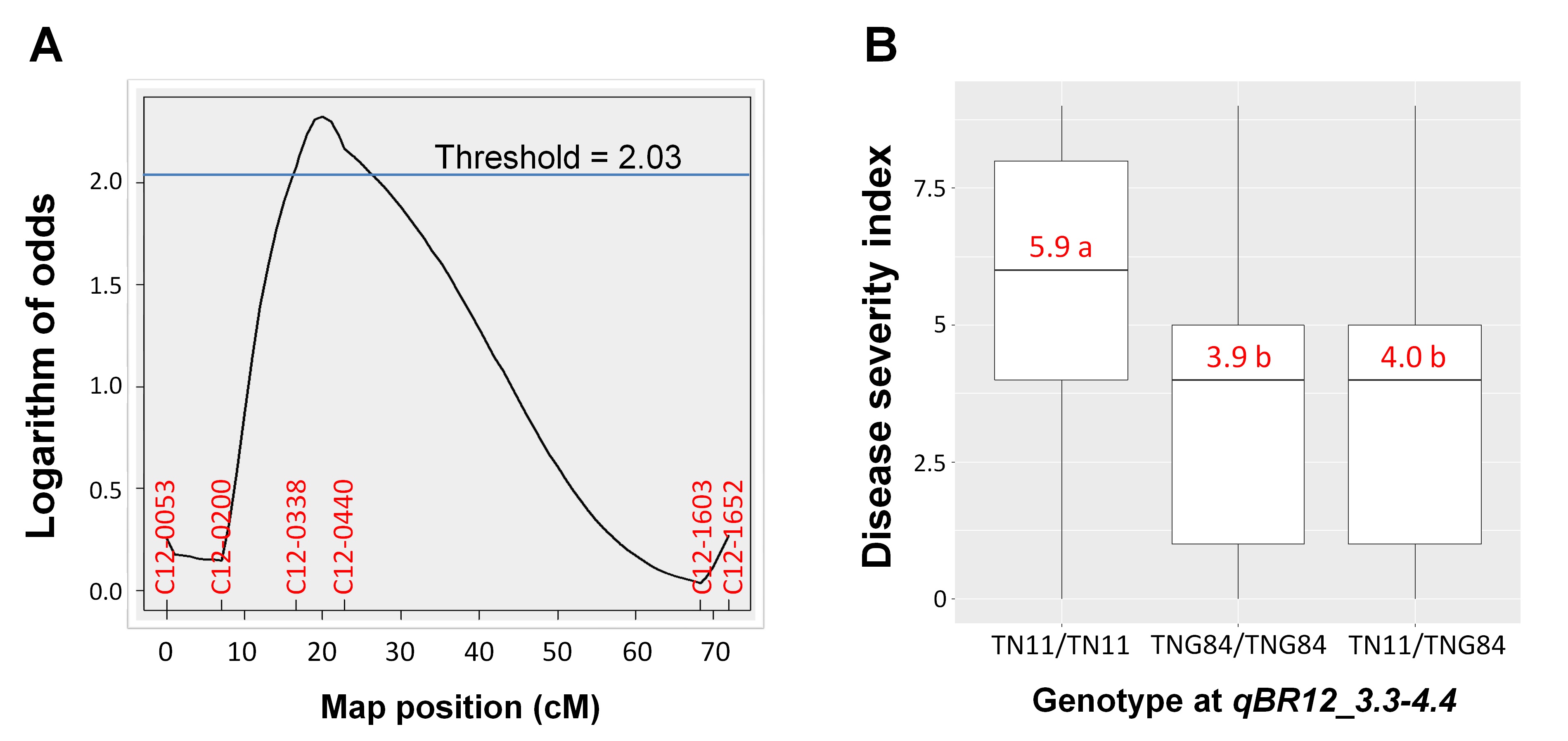


**Fig. S3** Analysis of resistance QTL against *P. oryzae* 12YL-TT4-1-lab in the BC_1_F_2_ population derived from the F_5:6_ line P092. (A) Logarithm of odds (LOD) scores between the markers C12_0053 (533,382 bp) and C12_1652 (16,527,453 bp) on chromosome 12. The horizontal line represents the LOD threshold at 95% confidence level based on 1,000 permutations. (B) Boxplot of the disease severity index (DSI) scores of different genotypes at *qBR12_3.3-4.4*. Data (mean) with different letters are significantly different according to Tukey’s HSD at *P* < 0.05. TN11/TN11 represents the homozygous genotype of the susceptible parent Tainan 11 (TN11) at *qBR12_3.3-4.4*; TNG84/TNG84 represents the homozygous genotype of the resistant parent Tainung 84 (TNG84); and TN11/TNG84 represents the heterozygous genotype.


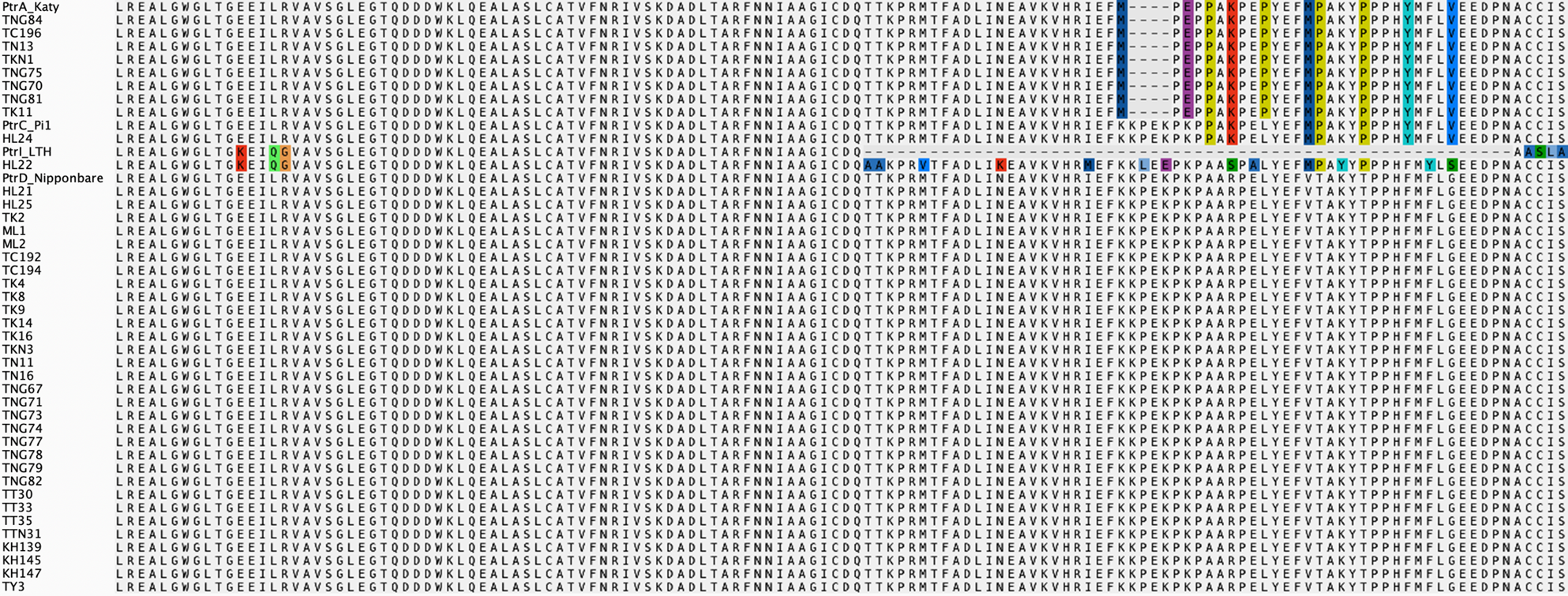


**Fig. S4** Amino acid sequences of the exon 4 of *Ptr* in 41 Taiwanese *japonica* rice cultivars. The cultivars included Tainung 84 (TNG84), Taichung 196 (TC196), Tainan 13 (TN13), Taikeng Glutinous 1 (TKN1), Tainung 75 (TNG75), Tainung 70 (TNG70), Tainung 81 (TNG81), Taikeng 11 (TK11), Hualien 24 (HL24), Hualien 22 (HL22), Hualien 21 (HL21), Hualien 25 (HL25), Taikeng 2 (TK2), Miaoli 1 (ML1), Miaoli 2 (ML2), Taichung 192 (TC192), Taichung 194 (TC194), Taikeng 4 (TK4), Taikeng 8 (TK8), Taikeng 9 (TK9), Taikeng 14 (TK14), Taikeng 16 (TK16), Taikeng Glutinous 3 (TKN3), Tainan 11 (TN11), Tainan 16 (TN16), Tainung 67 (TNG67), Tainung 71 (TNG71), Tainung 73 (TNG73), Tainung 74 (TNG74), Tainung 77 (TNG77), Tainung 78 (TNG78), Tainung 79 (TNG79), Tainung 82 (TNG82), Taitung 30 (TT30), Taitung 33 (TT33), Taitung 35 (TT35), Taitung Glutinous 31 (TTN31), Kaohsiung 139 (KH139), Kaohsiung 145 (KH145), Kaohsiung 147 (KH147), and Taoyuan 3 (TY3). The variety Katy containing the *PtrA* haplotype, Pil containing the *PtrC* haplotype, Nipponbare containing the *PtrD* haplotype, and Lijiangxintuanheigu (LTH) containing the *PtrI* haplotype were included as the reference (Greenwood et al. 2024; Zhao et al. 2018).
